# TBX2 specifies and maintains inner hair and supporting cell fate in the Organ of Corti

**DOI:** 10.1038/s41467-022-35214-4

**Published:** 2022-12-09

**Authors:** Marina Kaiser, Timo H. Lüdtke, Lena Deuper, Carsten Rudat, Vincent M. Christoffels, Andreas Kispert, Mark-Oliver Trowe

**Affiliations:** 1 Institute of Molecular Biology, Hannover Medical School, Hannover, Germany; 2 Medical Biology, Amsterdam Reproduction & Development, Amsterdam University Medical Centers, Amsterdam, The Netherlands

**Keywords:** Differentiation, Pattern formation, Auditory system

## Abstract

The auditory function of the mammalian cochlea relies on two types of mechanosensory hair cells and various non-sensory supporting cells. Recent studies identified the transcription factors INSM1 and IKZF2 as regulators of outer hair cell (OHC) fate. However, the transcriptional regulation of the differentiation of inner hair cells (IHCs) and their associated inner supporting cells (ISCs) has remained enigmatic. Here, we show that the expression of the transcription factor TBX2 is restricted to IHCs and ISCs from the onset of differentiation until adulthood and examine its function using conditional deletion and misexpression approaches in the mouse. We demonstrate that TBX2 acts in prosensory progenitors as a patterning factor by specifying the inner compartment of the sensory epithelium that subsequently gives rise to IHCs and ISCs. Hair cell-specific inactivation or misexpression causes transdifferentiation of hair cells indicating a cell-autonomous function of TBX2 in inducing and maintaining IHC fate.

## Abstract (web summary)

Inner hair cells are essential for hearing but the molecular drivers of their differentiation have remained enigmatic. Here, the authors show that the transcription factor TBX2 has a key function in inducing and maintaining inner hair cell fate.

## Introduction

**Fig. 1:**
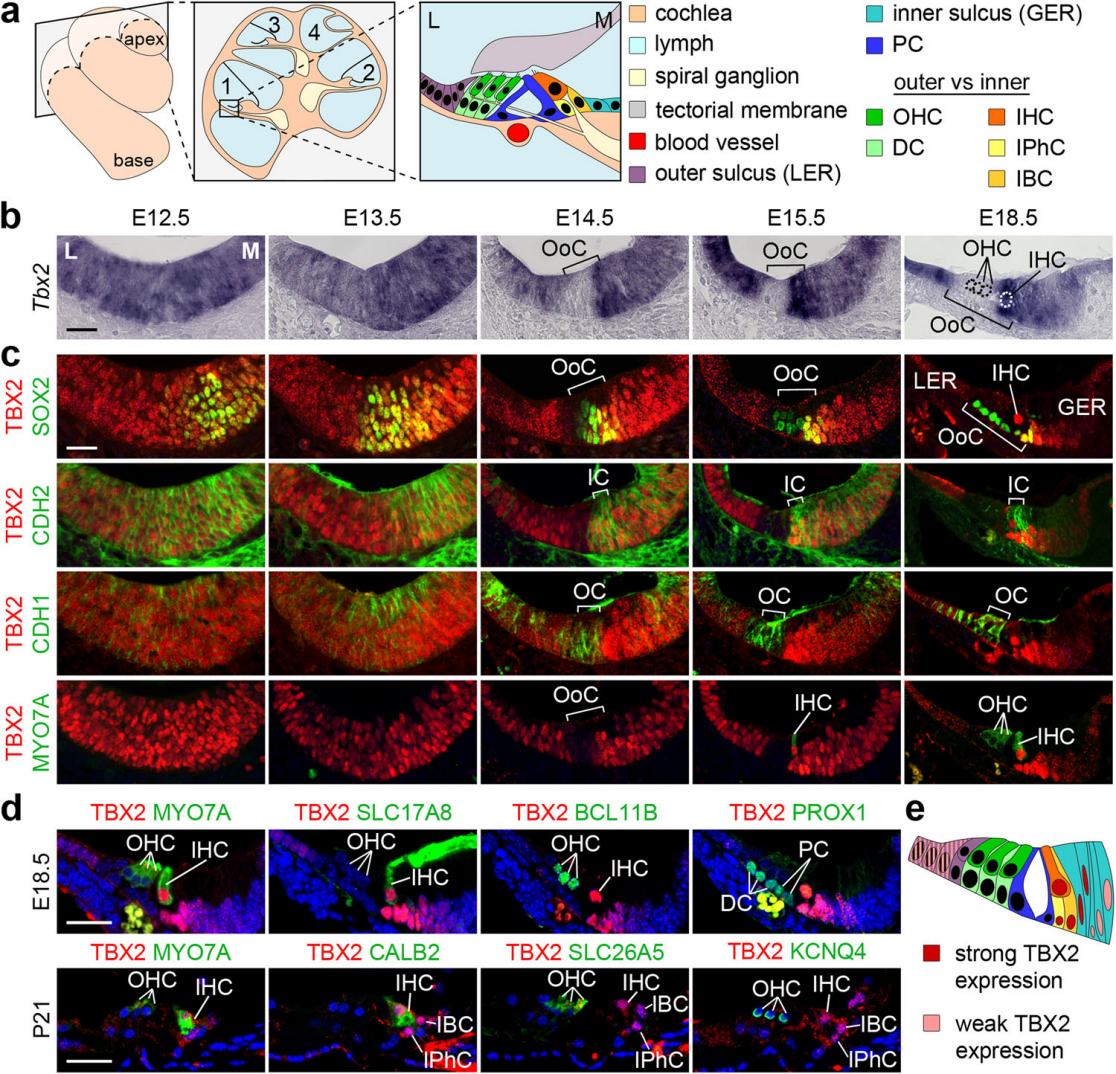
*Tbx2*/TBX2 expression is progressively confined to IHCs and ISCs during cochlear development. **a** Scheme showing the basal (1) and the apical (4) turn of the mature cochlea, a cross section through the cochlea, and a magnification of the basal turn with the cellular organization in and around the organ of Corti (OoC): with Deiters’ cells (DCs) and OHCs in the outer compartment (OC, shades of green), inner phalangeal cells (IPhCs), inner border cells (IBCs) and IHCs in the inner compartment (IC, shades of yellow) separated by pillar cells (PCs) that form the tunnel of Corti. Laterally, the OoC is flanked by cells of the outer sulcus, and medially by cells of the inner sulcus. *GER* greater epithelial ridge (primordium of the inner sulcus), *L* lateral, *LER* lesser epithelial ridge (primordium of the outer sulcus), *M* medial. **b**–**d** Expression of *Tbx2* mRNA and TBX2 protein on sections of the developing cochlea of wildtype embryos. **b** RNA in situ hybridization analysis of *Tbx2* expression in the prosensory epithelium of the cochlea at E12.5 and E13.5 as well as in the developing OoC between E14.5 and E18.5. Dotted circles mark the nucleus of the three OHCs and of one IHC in the OoC at E18.5. **c**, **d** Co-immunofluorescence analysis of expression of TBX2 with markers for differentiation (SOX2: pro-sensory cells/supporting cells; MYO7A: hair cells) and compartmentalization (CDH2: inner; CDH1: outer) from E12.5 to E18.5 (**c**) and with markers for all hair cells (MYO7A), IHCs (SLC17A8, CALB2), OHCs (BCL11B, SLC26A5, KCNQ4), DCs and PCs (PROX1) at E18.5 and P21 (**d**). *n* = 3 for each assay at each stage. Scale bars: 30 µm. **e** Scheme of TBX2 expression (red nuclei) in the OoC at E18.5. The color code is the same as in **a**.

Hearing relies on two types of highly specialized mechanosensory cells in the Organ of Corti, the sensory epithelium of the mammalian cochlea. Inner hair cells (IHCs) are the main sensory cells since they convert sound into auditory information; outer hair cells (OHCs) act as mechanical amplifiers that enhance sensitivity to sound and adjust frequency selectivity. IHCs and OHCs are not only functionally diverse, they also differ in size, shape, innervation, and number, and associate with distinct non-sensory supporting cells in separate compartments in the organ of Corti. A single row of large IHCs associates with two types of inner supporting cells (ISCs: inner phalangeal and inner border cells) in the inner compartment; smaller OHCs form three rows which are each supported by outer supporting cells (OSCs: Deiters’ cells) in the outer compartment. The two compartments are separated by two pillar cells that flank the fluid-filled tunnel of Corti^1,2^ (Fig. 1a).

All hair and supporting cells arise from common SOX2^+^ prosensory progenitors in embryonic development^3^. Activation of the transcription factor ATOH1 in a subset of these progenitors triggers hair cell differentiation, while Notch-mediated lateral inhibition prevents upregulation of ATOH1 in surrounding prosensory cells which subsequently differentiate into supporting cells^4,5^. Only at postnatal stages, hair cell differentiation is completed and IHCs and OHCs become functional^6^. Since mature cochlear hair cells cannot be regenerated^7^, their progressive loss due to age, excessive sound, or ototoxic drugs contributes to (age-related) deafness. Cell-based strategies may enable targeted regenerative therapies in the future but require the knowledge of factors driving hair cell diversification.

Recent work identified IKZF2 (Helios) and INSM1 as transcriptional regulators of OHC differentiation in the mouse^8,9^. IKZF2 is expressed in OHCs starting from postnatal day (P) 4. It is required for OHC maturation and is sufficient to induce an OHC-like fate in IHCs^8^. INSM1 is transiently expressed in OHCs at fetal and early postnatal stages (embryonic day (E) 15.5 - P2)^10^. Loss of this factor leads to transdifferentiation of a subset of OHCs into IHC-like cells with concomitant derepression of a set of IHC-specific genes including the T-box transcription factor gene *Tbx2*^9^. Since TBX2 acts as a patterning and differentiation factor in many developmental contexts^11^, including the anterior-ventral restriction of the neurogenic domain in the early otocyst^12^, it presents a strong candidate for an as yet unknown regulator of IHC differentiation.

Here, we characterize the dynamic expression of *Tbx2*/TBX2 during cochlear development. Using conditional deletion and misexpression approaches, we analyse the function of *Tbx2* in murine sensory cell development in a time-resolved manner. We show that prior to the onset of differentiation, TBX2 acts as a patterning factor for the inner compartment of the Organ of Corti from which IHCs and ISCs arise. At later stages TBX2 acts in a cell-autonomous manner to specify and maintain the IHC fate.

## Results and discussion

### TBX2 expression progressively restricts to IHCs and ISCs during cochlear development

To determine the expression pattern of *Tbx2* mRNA and TBX2 protein during cochlear development, we performed mRNA in situ hybridization and (co-)immunofluorescence analyses from cochlear outgrowth (E12.5) to a stage when hair cells have reached full maturity (P21) (Fig. 1b–e). At E12.5 and E13.5, *Tbx2* was widely expressed in the floor of the cochlear duct. Starting at E14.5, expression was excluded from the outer (lateral) region of the developing organ of Corti and was strongly upregulated in the inner (medial) region until E18.5. Weaker *Tbx2* expression was detected in cells of the adjacent greater and lesser epithelial ridges (Fig. 1b). TBX2 protein followed the pattern of the mRNA. Regionalisation of TBX2 expression in the developing organ of Corti succeeded the specification of prosensory cells (marked by SOX2) at E12.5 and E13.5, coincided spatially and temporarily with the formation of CDH1^+^ outer and CDH2^+^ inner compartments at E14.5, and preceded the differentiation of hair cells (marked by MYO7A) at E15.5 (Fig. 1c). Co-immunofluorescence analysis using specific markers for all hair cells (MYO7A), IHCs (SLC17A8, also known as VGLUT3; CALB2), OHCs (BCL11B; SLC26A5, also known as PRESTIN; KCNQ4), Deiters’ and pillar cells (PROX1) revealed that TBX2 is confined to IHCs and ISCs at E18.5 and P21 (Fig. 1d, e). This expression profile is compatible with a role for TBX2 in establishing and/or maintaining hair and supporting cells in the inner compartment of the organ of Corti.

### TBX2 mediates differentiation of IHCs and ISCs from prosensory progenitors

To explore the functional significance of compartmentalisation of TBX2 expression in the early development of the organ of Corti, we inactivated and misexpressed *Tbx2* in the prosensory domain at E12.5, using a tamoxifen-inducible *Sox2*^*CreERT2*^ mouse line^13^ in combination with a *loxP*-flanked *Tbx2* loss-of-function allele (*Tbx2*^*fl*^)^14^ and a *TBX2* misexpression allele which harbours a Cre-activatable TBX2-IRES-GFP expression cassette in the X-chromosomal *Hprt* locus (*Hprt*^*TBX2*^)^15,16^, respectively. Analysis of expression of a GFP reporter as well as of TBX2 protein confirmed efficient recombination and loss of TBX2 in the inner compartment of the organ of Corti in *Sox2*^*CreERT2/+*^*;Tbx2*^*fl/fl*^ (*Sox2-Tbx2LOF*) embryos and ectopic TBX2 expression at physiological levels in the medial half of the outer compartment in *Sox2*^*CreERT2/+*^*;Hprt*^*TBX2/Y(+)*^ (*Sox2-TBX2ME*) embryos as early as E14.5 (Supplementary Fig. 1).

**Fig. 2:**
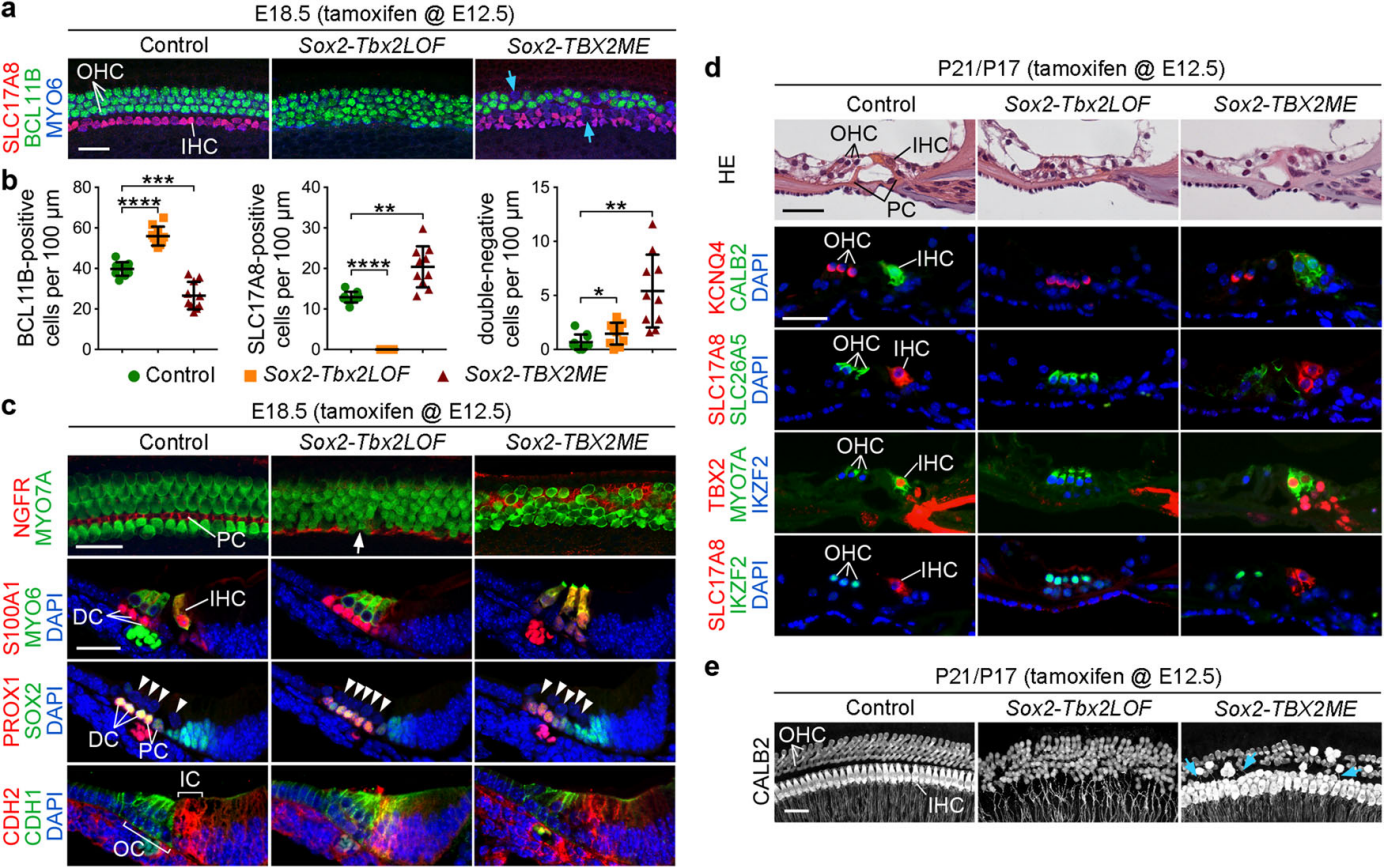
*Tbx2* regulates the differentiation of cells in the inner compartment of the Organ of Corti. **a**–**e** Analysis of hair and supporting cell differentiation in mice at E18.5 (**a**–**c**) and P21/P17 (**d**, **e**) in which *Tbx2* was ablated (*Sox2-Tbx2LOF*) or misexpressed (*Sox2-TBX2ME*) in prosensory cells by tamoxifen administration at E12.5. Note that we used male *Sox2-TBX2ME* mice, which misexpress TBX2 homogenously, for analysis at E18.5, and female *Sox2-TBX2ME* mice, which express the transgene in a mosaic fashion due to random X-chromosome inactivation, for analysis at P17. **a**, **b** Co-immunofluorescence analysis of expression of SLC17A8 (marks IHCs), BCL11B (marks OHCs) and MYO6 (marks all hair cells) on E18.5 whole cochleae (**a**) and quantification (**b**). Mean ± standard deviation, two-sided unpaired t-test with Welch’s correction or Mann-Whitney test. *p < 0.05 **p < 0.01; ***p < 0.001; ****p < 0.0001. *n* = 10 for each genotype. Blue arrows in **a** point to double-negative hair cells (SLC17A8^-^/BCL11B^-^). **c** Immunofluorescence analysis with antibodies against MYO7A/MYO6 (mark all hair cells), NGFR (pillar cells (PCs)), S100A1 (Deiters’ cells (DCs) and IHCs), PROX1 (DCs and PCs), SOX2 (all supporting cells), CDH1 (the outer compartment (OC)), CDH2 (the inner compartment (IC)) on whole cochleae (first row) and cochlear sections (second to fourth row) at E18.5. Arrow points to a gap in the shifted row of NGFR^+^ pillar cells in *Sox2-Tbx2LOF* embryos. Arrowheads point to the nuclei of hair cells. *n* = 6 for each genotype. Nuclei were counterstained with DAPI. **d** Histological staining (first row) and immunofluorescence analysis (second to fifth row) of markers for all hair cells (MYO7A), OHCs (KCNQ4, SLC26A5, IKZF2) and IHCs (CALB2, SLC17A8, TBX2) on cochlear sections of P21 control (*n* = 3), P21 *Sox2-Tbx2LOF* (*n* = 4) and P17 *Sox2-TBX2ME* mice (*n* = 2; the number of these mice was limited due to genetic burden). **e** Analysis of hair cell innervation by CALB2 immunofluorescence at the apical level of the organ of Corti of control (P21, *n* = 5), *Sox2-Tbx2LOF* (P21, *n* = 5) and *Sox2-TBX2ME* mutants (P17, *n* = 2). CALB2 marks type I afferent fibers, IHCs (strong), and OHCs (weak). Blue arrows point to fibers innervating ectopic IHC-like cells located in the outer compartment of the organ of Corti. Scale bars: 30 µm. The exact *p*-values and related source data are provided as a Source Data file.

We started the phenotypic characterization of hair and supporting cells in mutant cochleae at E18.5. In both *Sox2-Tbx2LOF* and *Sox2-TBX2ME* mutants the number of hair cells was preserved (Supplementary Fig. 2a) but they were misaligned in rows of four to five cells of width without a clear separation into one inner and three outer rows. In *Sox2-Tbx2LOF* embryos, expression of the IHC marker SLC17A8 was absent, while expression of the OHC marker BCL11B and the OHC regulator *Insm1*^9^ expanded to the innermost row of hair cells (Fig. 2a, b, Supplementary Fig. 2b). In contrast, in *Sox2-TBX2ME* cochleae the number of BCL11B^+^ OHCs was reduced while that of SLC17A8^+^ cells was increased paralleling the pattern of forced TBX2 expression (Fig. 2a, b, Supplementary Fig. 1f–h). In both transgenic conditions, a small number of hair cells lacked both SLC17A8 and BCL11B (Fig. 2a, b) indicating that these cells have not (yet) differentiated into one of the two hair cell subtypes.

The pattern of supporting cells followed the hair cell changes. In *Sox2-Tbx2LOF* cochleae, pillar cells (marked by NGFR) were shifted to border the innermost hair cell row. Moreover, all supporting cells underneath the hair cells strongly expressed S100A1 (marks Deiters’ cells) and PROX1 (marks Deiters’ and pillar cells), indicating a gain of OSCs at the expense of ISCs. In *Sox2-TBX2ME* mutants, NGFR^+^ pillar cells intermingled with OHC rows. Expression of PROX1 and S100A1 was strongly reduced indicating a gain of ISCs at the expense of OSCs. Hair and supporting cell changes in *Sox2-Tbx2LOF* and *Sox2-TBX2ME* embryos were accompanied by a medial expansion and reduction, respectively, of the outer compartment marker CDH1 (Fig. 2c, Supplementary Fig. 2c), pointing to disturbed compartmentalization of the organ of Corti at this stage.

Histological and molecular analysis of mice at P21 and P17, respectively, revealed that the altered patterns of hair cell types observed at E18.5 were preserved in mature *Sox2-Tbx2LOF* and *Sox2-TBX2ME* cochleae. In *Sox2-Tbx2LOF* mice, all cochlear hair cells presented features of OHCs (smaller size, smaller nucleus, expression of KCNQ4 and SLC26A5). Importantly, these cells also expressed IKZF2, the key regulator of OHC maturation. In contrast, *Sox2-TBX2ME* cochleae exhibited an increased number of hair cells with characteristics of IHCs (larger size, large nucleus, expression of SLC17A8 and CALB2) while the number of (IKZF2^+^) OHCs was correspondingly decreased (Fig. 2d). Because IHCs and OHCs differ in their neural innervation, we stained for CALB2, which labels type I afferent neurons of the spiral ganglion that exclusively contact IHCs. In *Sox2-Tbx2LOF* cochleae, the number of CALB2^+^ afferent fibers innervating the OHC-like cells in the region of the former IHC compartment was strongly reduced. 23% (168 out of 763) of the OHCs and OHC-like cells were innervated. In *Sox2-TBX2ME* cochleae, 92% (195 out of 211) of the ectopic IHC-like cells were innervated by CALB2^+^ afferents (Fig. 2e).

Together these data show that in prosensory progenitors *Tbx2* is both required and sufficient to regulate differentiation of ISCs and correctly innervated IHCs.

### TBX2 patterns prosensory progenitors in an inner and an outer compartment

**Fig. 3:**
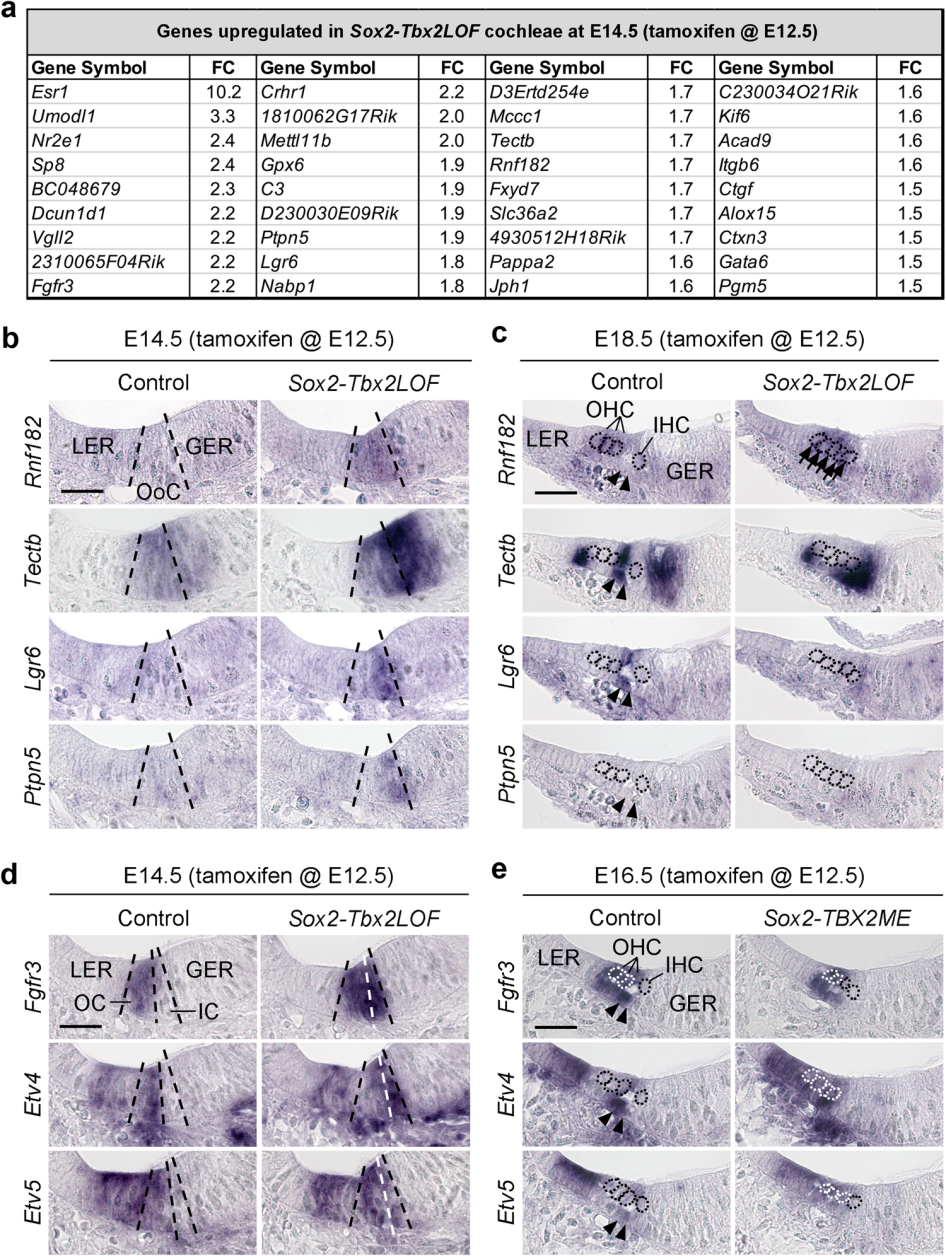
*Tbx2* patterns prosensory progenitors in the developing organ of Corti. **a**–**e** Expression analysis of genes with increased expression in microarrays of E14.5 cochleae in which *Tbx2* was ablated in prosensory cells (*Sox2-Tbx2LOF*) by tamoxifen administration at E12.5. **a** List of genes with increased expression in E14.5 *Sox2-Tbx2LOF* cochleae as detected by microarray analysis (*n* = 4). FC, fold change **b**–**e** RNA in situ hybridization analysis of selected candidates (**b**, **c**) on E14.5 (**b**) and E18.5 (**c**) cochlear sections of *Sox2-Tbx2LOF* embryos, and of *Fgfr3* and its target genes *Etv4* and *Etv5* (**d**, **e**) on cochlear sections of E14.5 *Sox2-Tbx2LOF* (**d**) and E16.5 *Sox2-TBX2ME* (**e**) embryos. *n* = 3 for each genotype at each stage. Arrows in **c** point to *Rnf182*-expressing hair cells, arrowheads to pillar cells (PCs). Dashed lines in **b**, **d** show the position of the developing organ of Corti and the subdivision into the inner compartment (IC) and outer compartment (OC). Dotted circles in **c**, **e** mark the nuclei of hair cells. Scale bars: 30 µm. *LER* lesser epithelial ridge, *GER* greater epithelial ridge, *OoC* organ of Corti.

The disturbed compartmentalization in *Sox2-Tbx2LOF* mice may result from a mispatterning of prosensory progenitors or from a transdifferentiation of hair and supporting cells that have been correctly specified. To discriminate between these two possibilities, we performed transcriptional profiling by microarray analysis of control and mutant cochlear ducts at E14.5 when hair and supporting cell differentiation has not yet occurred. *Significance Analysis of Microarrays* (SAM) identified 36 upregulated and 90 downregulated genes in the mutant. Among the list of downregulated transcripts, some genes have been described to be expressed in cells of the inner compartment such as *Pvalb*^17^ and *Fgf8*^18^, *Fabp7*^19^, and *Lfng*^20^ (Supplementary Table 1). In contrast, some of the upregulated genes were previously identified as markers of the outer compartment (*Crhr1*^21,22^, *Lgr6*^23^, *Ctgf*^24^, and *Fgfr3*^25^) (Fig. 3a, Supplementary Table 2) indicating an expansion of the outer at the expense of the inner compartment at onset of organ of Corti development.

This notion was corroborated by in situ hybridization analysis of upregulated genes in E14.5 and E18.5 *Sox2-Tbx2LOF* cochleae. *Rnf182*, a gene expressed in OHCs of control animals at E18.5, was ectopically expressed in the entire developing organ of Corti at E14.5. Expression of *Tectb* was strongly enhanced in the inner compartment of the organ of Corti and in the adjacent greater epithelial ridge. *Lgr6* and *Ptpn5* were ectopically expressed in the inner compartment (Fig. 3b, c). Since *Lgr6* marks pillar cells starting from E15.5^23^, this points to a premature specification of this cell type. Together with loss of CDH2 expression in the inner compartment of the developing organ of Corti at E14.5 (Supplementary Fig. 3), these changes indicate that the inner compartment is not established in the prosensory region of *Sox2-Tbx2LOF* embryos.

We noted increased and/or ectopic expression of some candidate genes in the cochlear epithelium outside the organ of Corti in *Sox2-Tbx2LOF* embryos (Supplementary Fig. 4) pointing to a functional implication of TBX2 expression in the lesser and greater epithelial ridges.

*Fgfr3* (fibroblast growth factor receptor 3) was the only deregulated gene, which exhibited a complementary pattern of expression to *Tbx2* in the developing organ of Corti. *Fgfr3* was expressed in the outer compartment of control cochleae. Its expression expanded into the inner compartment in *Sox2-Tbx2LOF* embryos and was medially reduced in *Sox2-TBX2ME* mutants. Concordantly, expression of *Etv4* and *Etv5*, direct targets of FGF signaling^26^, was medially expanded upon *Tbx2*-deletion and the *Etv4/5*-negative outer region was lost upon *TBX2*-misexpression (Fig. 3d, e).

Previous work implicated FGF signalling in multiple aspects of cell differentiation in the outer compartment of the organ of Corti^25,27–31^. Notably, lineage tracing experiments in mouse demonstrated that OHCs, OSCs and pillar cells derive from FGFR3^+^ progenitors^32^, and deletion or inactivation of *Fgfr3* resulted in disturbed maturation of pillar cells and reduced expression of the OHC-marker SLC26A5^18,33^. This strongly suggests that *Tbx2* establishes the inner compartment of the organ of Corti by preventing FGFR3-dependent FGF signalling in this region. Given numerous reports that TBX2 acts as a transcriptional repressor in a wide variety of developmental contexts^34^, early compartmentalization of the organ of Corti may rely on direct repression of *Fgfr3* expression by TBX2.

### TBX2 maintains the fate of differentiating IHCs and ISCs

**Fig. 4:**
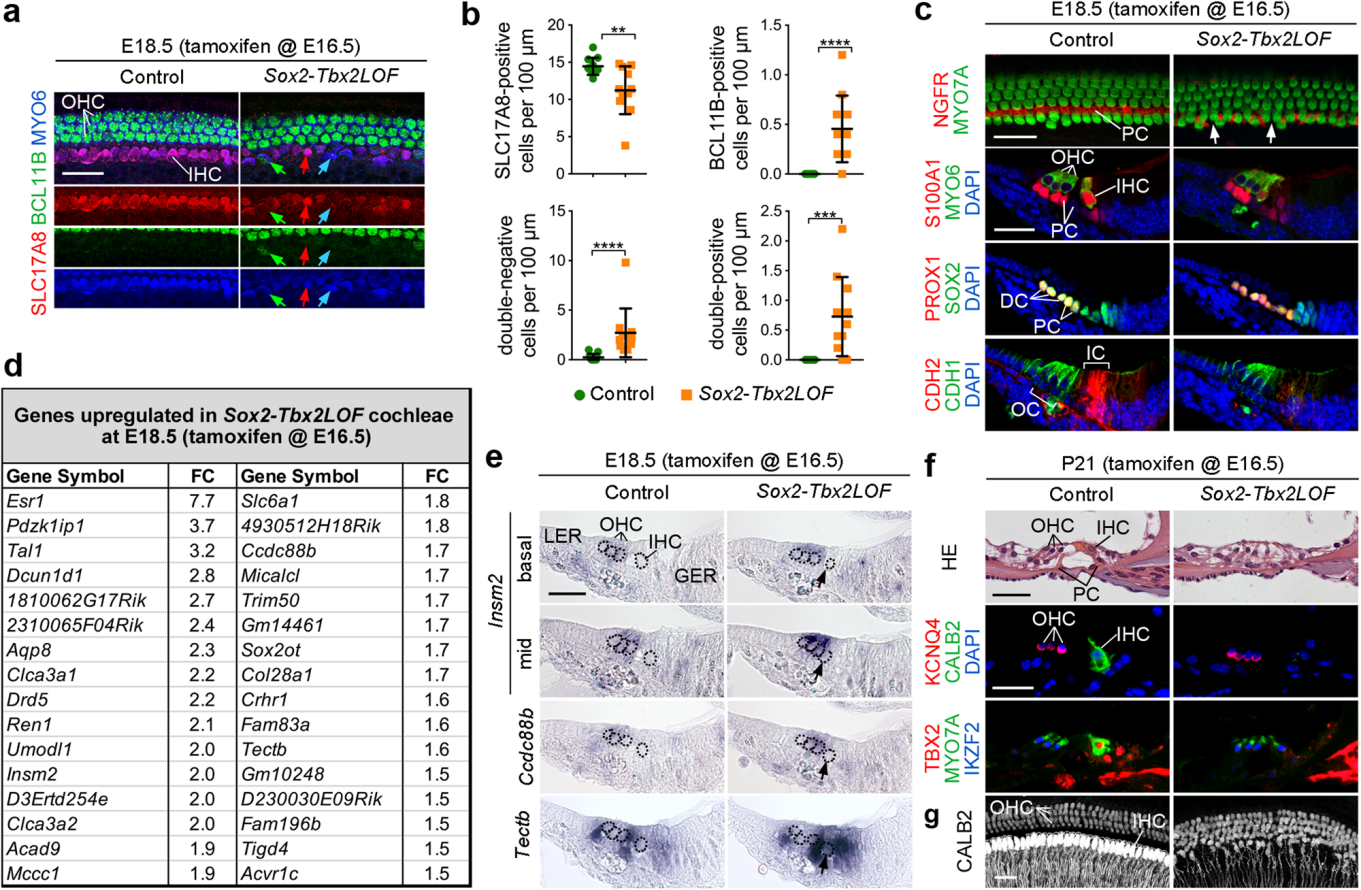
*Tbx2* maintains IHC and ISC fate by preventing transdifferentiation into OHCs and OSCs, respectively. **a**–**d** Hair and supporting cell differentiation in mice in which *Tbx2* was ablated (*Sox2-Tbx2LOF*) in SOX2^+^ cells in the organ of Corti by tamoxifen administration at E16.5. **a**, **b** (Co-)immunofluorescence analysis of IHC and OHC differentiation in E18.5 whole cochleae by antibodies against MYO6 (all hair cells), BCL11B (OHCs), SLC17A8 (IHCs). Red arrow points to a MYO6^+^/SLC17A8^+^ IHC, green arrow to a MYO6^+^/BCL11B^+^ OHC-like and blue arrow to a double-negative hair cell (SLC17A8^-^/BCL11B^-^) in the innermost hair cell row (**a**). **b** Quantification of hair cell types in the inner compartment. Mean±standard deviation, two-sided unpaired t-test with Welch’s correction or Mann-Whitney-U test. **p < 0.01; ***p < 0.001; ****p < 0.0001. *n* = 11 for each genotype. **c** (Co-)immunofluorescence analysis of supporting cell differentiation in E18.5 cochleae by antibodies against MYO7A/MYO6 (all hair cells), NGFR (pillar cells (PCs)), S100A1 (Deiters’ cells (DCs) and IHCs), PROX1 (DCs and PCs), SOX2 (all supporting cells) and of compartmentalization by antibodies against CDH1 in the outer compartment (OC) and CDH2 in the inner compartment (IC). Nuclei were counterstained with DAPI. Arrows points to gaps in the shifted and irregular row of NGFR^+^ pillar cells. *n* = 6 for each genotype. **d** List of genes with increased expression in E18.5 *Sox2-Tbx2LOF* organs of Corti as detected by microarray analysis (*n* = 4). FC, fold change. **e** Validation of selected microarray candidates by RNA in situ hybridization analysis. *Insm2* expression is shown at the basal and medial (mid) turn of the cochlear duct. Dotted circles mark the nuclei of hair cells. Arrow points to the innermost hair cell. *n* = 3 for each genotype. **f** Histological analysis (first row) and immunofluorescence (second and third row) of hair cell markers at P21: MYO7A (all hair cells), KCNQ4 and IKZF2 (OHCs), CALB2 and TBX2 (IHCs). *n* = 5 for each genotype. **g** Analysis of hair cell innervation by CALB2 immunofluorescence at the apical level of the organ of Corti of P21 control and *Sox2-Tbx2LOF* mice. CALB2 marks type I afferent fibers, IHCs (strong) and OHCs (weak). *n* = 5 for each genotype. Scale bars: 30 µm. *LER* lesser epithelial ridge, *GER* greater epithelial ridge. The exact *p*-values and related source data are provided as a Source Data file.

We next explored whether TBX2 is critical for maintaining the fate of cochlear hair and supporting cells. At E16.5, when these cell types are established at the base of the cochlear duct^9,10,18,35^,they still express SOX2. We therefore administered a single pulse of tamoxifen at E16.5 to *Sox2-Tbx2LOF* embryos and analysed phenotypic changes at E18.5 (Fig. 4a–c). Since we did not obtain *Sox2-TBX2ME* embryos at this stage, we limited our analysis to the loss-of-function situation.

In *Sox2-Tbx2LOF* cochleae, the total number of hair cells was unchanged (Supplementary Fig. 5a). Hair cells in the outer compartment of the organ of Corti were perfectly aligned and expressed the OHC marker BCL11B comparable to control. In contrast, hair cells of the inner compartment were misaligned, showed partly reduced expression of IHC markers SLC17A8 and S100A1, and to some extent ectopic expression of the OHC-marker BCL11B. NGFR^+^ pillar cells were intermingled with the innermost row of hair cells. Although the number of S100A1^+^ Deiters’ cells was unchanged, the PROX1^+^ OSC population expanded medially in *Sox2-Tbx2LOF* mutants. Expression of CDH1 was normal but CDH2 was strongly downregulated in the inner compartment (Fig. 4a–c).

Transcriptional profiling of E18.5 *Sox2-Tbx2LOF* cochlear ducts by microarray analysis identified 48 down- and 32 upregulated genes. Among the genes with reduced expression, we found candidates whose specific or enhanced expression in cells of the inner compartment was previously described: *Fgf8*^18^, *Cabp2*^36^, *Slc17a8*^37^ and *Gabrg3*^38^ in IHCs, *Fgf20*^27^ and *Otol1*^39^ in ISCs, and *Npy*^32^ in inner pillar cells (Supplementary Table 3). In the list of upregulated genes, some genes have been described to be expressed in cells of the outer compartment: *Pdzk1ip1*^32^, *Tal1*^22^, *Drd5*^40^, *Insm2*^9^, and *Crhr1*^21^ (Fig. 4d, Supplementary Table 4). Only very few candidates were detectable by RNA in situ hybridization in control and mutant embryos at E18.5. *Trh*, *Il33* and *Otol1* were expressed in the inner domain of the organ of Corti and were downregulated in the mutant (Supplementary Fig. 5b). *Insm2* and *Ccdc88* showed increased expression in OHCs but were not ectopically expressed in the innermost hair cell indicating non-cell-autonomous effects of *Tbx2* loss in the outer compartment. *Tectb* was enhanced in the inner compartment and the greater epithelial ridge (Fig. 4e). *Pdzk1ip1*, *Tal1* and *Slc6a1* were expressed in Deiters’ cells in both control and mutant (Supplementary Fig. 5c). Together, marker analysis and transcriptional profiling suggest that in E18.5 *Sox2-Tbx2LOF* cochleae, hair and supporting cells of the inner compartment have undergone a partial fate shift.

At P21, almost all hair cells expressed OHC-specific markers (IKZF2, SLC26A5, KCNQ4) whereas expression of IHC-specific markers (CALB2, SLC17A8) was not detected (Fig. 4f, Supplementary Fig. 5d). Moreover, CALB2^+^ afferent fibers that normally innervate IHCs, were strongly reduced (Fig. 4g) indicating a complete conversion of IHCs to OHCs after an extended time interval. This shows that *Tbx2* is required to maintain the fate of IHCs and ISCs after E16.5 by preventing their transdifferentiation into OHC and OSC, respectively.

To address whether FGFR3-mediated signalling contributes to the observed cytodifferentiation defects in *Sox2-Tbx2LOF* embryos, we analysed the expression of some FGF signalling components at E18.5. Hair cells of the inner row lacked expression of *Fgf8* consistent with the loss of an IHC fate. Expression of *Fgfr3* was medially expanded but did not translate into expression of the FGF targets *Etv4* and *Etv5* that mark pillar cells at this stage (Supplementary Fig. 5e). This precludes transcription-dependent effects of FGFR3 signalling as contributors of hair and supporting cell fate changes in this setting.

### TBX2 cell-autonomously induces IHC fate in nascent hair cells

**Fig. 5:**
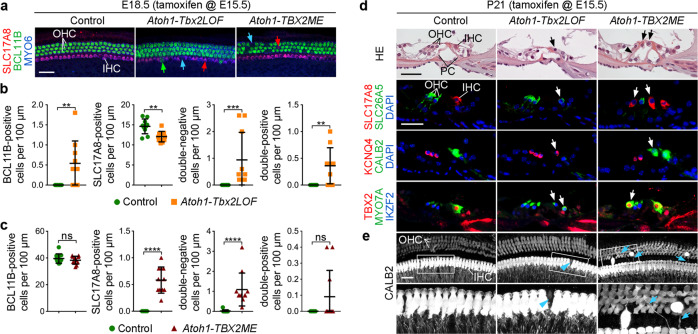
TBX2 cell-autonomously induces IHC fate in nascent hair cells. **a**–**e** Analysis of hair cell differentiation in E18.5 embryos (**a**–**c**) and P21 mice (**d**, **e**) in which *Tbx2* was ablated (*Atoh1-Tbx2LOF*) or misexpressed (*Atoh1-TBX2ME*) in individual hair cells by tamoxifen administration at E15.5. **a**–**c** (Co-)immunofluorescence analysis of IHC and OHC differentiation in E18.5 whole cochleae by antibodies against MYO6 (all hair cells), BCL11B (OHCs), SLC17A8 (IHCs). Red arrow points to MYO6^+^/SLC17A8^+^, green arrow to a MYO6^+^/BCL11B^+^ and blue arrow to a double-negative (SLC17A8^-^/BCL11B^-^) hair cell (**a**). (For single channels see Supplementary Fig. 7b). **b**, **c** Quantification of hair cell types in the inner row of hair cells in *Atoh1-Tbx2LOF* (**b**) and in the three outer hair cell rows in *Atoh1-TBX2ME* cochleae (**c**). Mean ± standard deviation, two-sided unpaired t-test or Mann-Whitney-U test. ns, not significant; **p < 0.01; ***p < 0.001; ****p < 0.0001. *n* = 10 (**b**) and *n* = 11 (**c**) for each genotype. **d** Histological analysis (first row) and immunofluorescent detection (second to fourth row) of markers for all hair cells hair (MYO7A), IHCs (SLC17A8, CALB2, TBX2) and OHCs (KCNQ4, SLC26A5, IKZF2) on cochlear sections of P21 control (*n* = 4), *Atoh1-Tbx2LOF* (*n* = 4) and *Atoh1-TBX2ME* mice (n = 7). Arrows (black in HE, white in immunodetections) point to ectopic OHCs and IHCs, respectively. Black arrowhead points to an ectopic pillar cell (PCs). Nuclei were counterstained with DAPI. **e** Analysis of hair cell innervation by CALB2 immunofluorescence at the apical level of the organ of Corti of P21 control (*n* = 5)*, Atoh1-Tbx2LOF* (*n* = 5) and *Atoh1-TBX2ME* (*n* = 7) mice. CALB2 marks type I afferent fibers, IHCs (strong) and OHCs (weak). Magnified regions are marked by rectangles. The blue arrowhead marks a hair cell in the inner row of *Atoh1-Tbx2LOF* cochleae, which lacks strong CALB2 staining (i.e., an ectopic OHC-like cell). Blue arrows point to fibers innervating hair cells strongly positive for CALB2 in the outer rows (i.e., ectopic IHC-like cells). Scale bars: 30 µm. The exact *p*-values and related source data are provided as a Source Data file.

To manipulate *Tbx2* function specifically in hair cells, we used an *Atoh1-CreERT2* mouse line^41^ that mediates stochastic recombination in hair cells at low frequency after tamoxifen administration at E15.5 or at P0-1 (Supplementary Fig. 6). We first investigated the phenotypic consequences of manipulating *Tbx2* expression by a single pulse of tamoxifen administration at E15.5, i.e., a stage when hair cells have just appeared. We found that the total number of MYO6^+^ hair cells and their separation in one inner and three outer rows was preserved in the cochlea of both *Atoh1-CreERT2/+;Tbx2*^*fl/fl*^ (*Atoh1-Tbx2LOF*) and *Atoh1-CreERT2/+;Hprt*^*TBX2/+*^ *(Atoh1-TBX2ME*) mutants at E18.5 (Supplementary Fig. 7a, Fig. 5a). However, in the innermost hair cell row of *Atoh1-Tbx2LOF* cochleae the number of hair cells expressing the IHC marker SLC17A8 was decreased whereas the number of hair cells expressing the OHC-marker BCL11B, or none or both of these markers was increased (Fig. 5a, b, Supplementary Fig. 7b). In *Atoh1-TBX2ME* embryos, we detected a significantly increased number of SLC17A8^+^ and BCL11B/SLC17A8-double-negative hair cells in the outer compartment of the organ of Corti (Fig. 5a, c, Supplementary Fig. 7b).

Since we found a fate switch of supporting cells upon early loss or misexpression of *Tbx2* in the prosensory domain, we investigated whether the subtype switch of individual hair cells leads to a corresponding switch of supporting cell fate by a non-cell autonomous mechanism (i.e., without altering *Tbx2* expression in supporting cells). Immunofluorescence analysis on whole-mount preparations of E18.5 organs of Corti revealed gaps in the row of NGFR^+^ pillar cells in *Atoh1-Tbx2LOF*, but no alteration in *Atoh1-TBX2ME* mutants. Expression of the OSC marker PROX1 and the ISC marker GLAST was unaffected in both mutants (Supplementary Fig. 7c). This indicates that the proper formation of the tunnel of Corti is susceptible to the locally restricted loss of IHCs but that conversion of hair cells is not sufficient to induce the conversion of the underlying supporting cells as previously reported^42^.

Histological analysis at P21 revealed a normal structural appearance of the organ of Corti in *Atoh1-Tbx2LOF* mutants. However, hair cells located medially to pillar cells, i.e., in the inner row, expressed OHC markers (SLC26A5, KCNQ4) and the regulator of OHC-fate IKZF2 instead of IHC markers (SLC17A8, CALB2). In contrast, in *Atoh1-TBX2ME* embryos, we found ectopic IHC-like cells and supernumerary pillar cells in the outer compartment of the organ of Corti (Fig. 5d, Supplementary Fig. 8). OHC-like cells in the inner row of *Atoh1-Tbx2LOF* mutants appeared to be innervated by CALB2^+^ type I afferents (13 out of 13). In *Atoh1-Tbx2ME* cochleae 50% (9 out of 18) of the ectopic IHC-like cells were innervated (Fig. 5e). Together, these data show that TBX2 confers IHC fate cell-autonomously and independently from positional information to nascent hair cells.

### Transdifferentiation of hair cells upon neonatal *Tbx2* manipulation

**Fig. 6:**
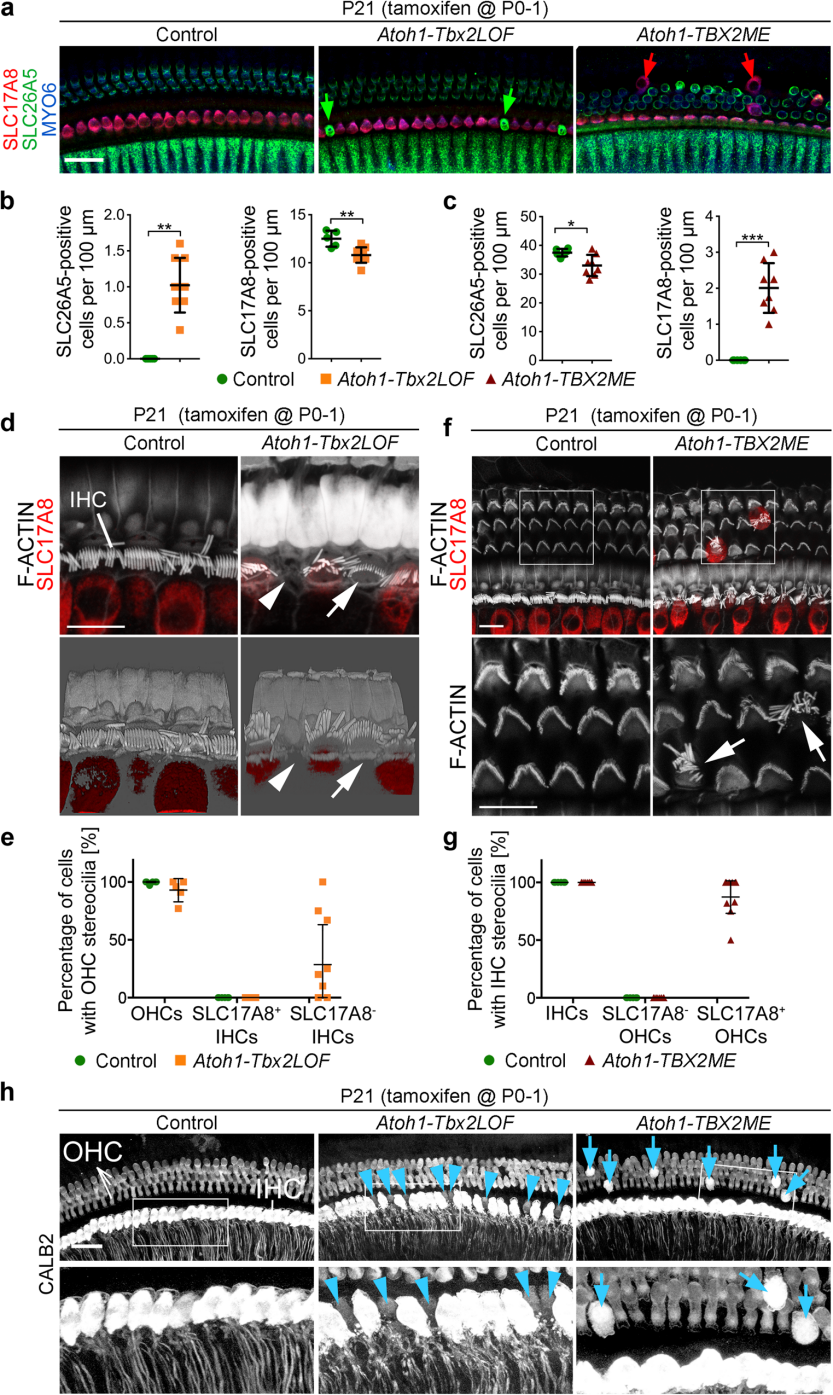
Postnatal *Tbx2* inactivation or misexpression causes cell-autonomous transdifferentiation of hair cells. **a**–**h** Analysis of hair cell differentiation in P21 mice in which *Tbx2* was ablated (*Atoh1-Tbx2LOF*) or misexpressed (*Atoh1-TBX2ME*) in individual hair cells by tamoxifen administration at P0-1. **a**–**c** Co-immunofluorescence analysis of hair cell differentiation with markers for all hair cells (MYO6), IHCs (SLC17A8) and OHCs (SLC26A5). Green arrows point to ectopic and small MYO6^+^/SLC26A5^+^ OHCs in the innermost hair cell row of *Atoh1-Tbx2LOF* mice. Red arrows point to ectopic and large MYO6^+^/SLC17A8^+^ IHCs in the outermost hair cell rows of *Atoh1-TBX2ME* mice. *n* = 10 for each genotype (**a**). **b**, **c** Quantification of SLC26A5^+^ and SLC17A8^+^ hair cells in the inner compartment of *Atoh1-Tbx2LOF* (n = 5 for controls, *n* = 9 for mutants) (**b**) and the outer compartment of *Atoh1-TBX2ME* (*n* = 6 for controls, n = 8 for mutants) (**c**) organs of Corti at the basal level. Mean±standard deviation, two-sided unpaired t-test or Mann-Whitney-U test. **, *p* < 0.01; ***p < 0.001. **d**–**g** Analysis of stereocilia bundles by immunofluorescence and 3D-reconstructions in organs of Corti of controls (*n* = 6), *Atoh1-Tbx2LOF* (*n* = 8) (**d**, **e**) and *Atoh1-TBX2ME* embryos (*n* = 12) (**f**, **g**). F-ACTIN was detected with fluorophore-conjugated-Phalloidin, IHCs by an antibody against SLC17A8. **d** Arrowheads point to SLC17A8^-^ hair cells without stereocilia, arrows to SLC17A8^-^ hair cells with OHC stereocilia bundle morphology in the inner compartment. **e** Quantification of hair cells with OHC bundle morphology. Weighted Mean±standard deviation. **f** Arrows point to SLC17A8^+^ hair cells with IHC stereocilia bundle morphology in the outer compartment. **g** Quantification of hair cells with IHC bundle morphology. Weighted Mean±standard deviation. **h** Analysis of hair cell innervation by CALB2 immunofluorescence at the apical level of the organ of Corti of control (*n* = 5), *Atoh1-Tbx2LOF* (*n* = 5) and *Atoh1-TBX2ME* (*n* = 10) mice. Blue arrowheads point to small CALB2-negative hair cells (i.e., OHC-like cells) in the inner row of the *Atoh1-Tbx2LOF* cochlea; blue arrows point to large CALB2-positive cells (i.e., IHC-like cells) in the outer rows of a *Atoh1-TBX2ME* cochlea. Note that these IHC-like cells are not innervated. Scale bars: 30 µm. The exact *p*-values and related source data are provided as a Source Data file.

We next inactivated or misexpressed *Tbx2* using the *Atoh1-CreERT2* mouse line and applying a single pulse of tamoxifen to breast feeding dams at P0-1. i.e., stage when differentiated IHCs and OHCs are established. Subsequently, the phenotypic consequences of manipulated *Tbx2* expression were analyzed at P21. The total number of hair cells in the organ of Corti was preserved in both *Atoh1-Tbx2LOF* and *Atoh1-TBX2ME* mice (Supplementary Fig. 9a) but in the innermost hair cell row of *Atoh1-Tbx2LOF* cochleae the number of SLC17A8^+^ hair cells was decreased whereas the number of SLC26A5^+^ hair cells was increased (Fig. 6a, b). Conversely, in *Atoh1-TBX2ME* embryos, we detected a decreased number of SLC26A5^+^ and increased number of SLC17A8^+^ hair cells in the outer compartment of the organ of Corti (Fig. 6a, c). In contrast to the analysis at E18.5, we did not find any SLC26A5/SLC17A8-double-positive or -negative hair cells (Supplementary Fig. 9b, c) indicating that non-committed hair cells differentiate to a specific hair cell type after birth.

IHCs and OHCs differ in stereocilia bundle morphology. Whereas OHCs have shorter stereocilia that are arranged in V-shaped bundles, IHC stereocilia are longer and arranged in linear bundles. Co-staining of F-ACTIN and SLC17A8 demonstrated that upon loss of *Tbx2* 29% of the SLC17A8^-^ IHCs had stereocilia with bundle morphology resembling that of OHCs whereas 71% of these hair cells had no stereocilia at all (Fig. 6d, e). This indicates that *Tbx2* is required to maintain IHC fate at postnatal stages, but that these *Tbx2*-deficient hair cells do not completely convert to OHCs. Upon misexpression of *TBX2* 87% of the SLC17A8^+^ OHCs (i.e., ectopic IHC-like cells) exhibited stereocilia with a bundle morphology resembling that of IHCs (Fig. 6f, g) indicating a robust conversion from OHC to IHC identity.

OHC-like cells in the inner row of *Atoh1-Tbx2LOF* cochleae were innervated by CALB2^+^ type I afferents (32 out of 32). In *Atoh1-Tbx2ME* cochleae, only 7% of the ectopic IHC-like cells (12 out of 162) were innervated by CALB2^+^ fibers indicating that innervation patterns of hair cells are stably established before birth (Fig. 6h). We conclude that neonatal *Tbx2* inactivation or misexpression causes cell-autonomous transdifferentiation of hair cells, which however, maintain their initial innervation pattern.

While we were in the process of revising this manuscript, a study reported that embryonic and neonatal differentiating IHCs acquire phenotypic characteristics of OHCs when *Tbx2* was conditionally deleted in these cells^43^. Our study confirms that *Tbx2* is required to prevent this fate switch but additionally uncovered that TBX2 is sufficient to induce complete OHC-to-IHC conversion upon ectopic activation during fetal stages, whereas a partial fate shift without proper innervation occurred when TBX2 was misexpressed postnatally.

Our study also found that the function of TBX2 as a differentiation factor is not limited to hair cells but extends to supporting cells. Moreover, we show that prior to cell differentiation TBX2 acts as a patterning factor by specifying the inner compartment of the organ of Corti, which subsequently gives rise to IHCs and ISCs. Our transcriptional profiling experiments suggest that *Fgfr3* is a critical target of TBX2 during its early patterning function, while its latter role in differentiated IHCs is possibly mediated by repression of regulators of OHC fate such as IKZF2.

Together, the two studies defined that TBX2 sets up the transcriptional network driving inner hair and supporting cell fate in the Organ of Corti. This finding paves the way to further unravel the molecular circuits of cochlear hair and supporting cell differentiation and rationalizes therapeutic avenues in hair cell-related hearing disorders.

## Methods

### Ethics and animals

Up to four mice per cage were housed with ad libitum access to food and water under conditions of regulated temperature (22 °C) and humidity (50%) and a 12-h light/ dark cycle at the central animal laboratory of Hannover Medical School. The experiments were in accordance with the German Animal Welfare Legislation and approved by the local Institutional Animal Care and Research Advisory Committee and permitted by the Lower Saxony State Office for Consumer Protection and Food Safety (Permit Number: 33.12-42502-04-19/3081).

All mouse alleles employed in this study have previously been described and were maintained on an NMRI genetic background. A conditional floxed allele of *Tbx2* [*Tbx2*^*tm2.1Vmc*^, synonym: *Tbx2*^*fl*^]^14^ was provided by Vincent Christoffels; an allele with insertion of the human *TBX2* gene at the *Hprt* locus [*Hprt*^*tm2(CAG-TBX2,-EGFP)Akis*^, synonym: *Hprt*^*TBX2*^]^15^ was generated in house. The double fluorescent Cre reporter line *Gt(ROSA)26Sor*^*tm4(ACTB-tdTomato,-EGFP)Luo/J*^ [#007576, synonym: *R26*^*mTmG*^]^44^, the tamoxifen-inducible *CreERT2* driver lines *Sox2*^*CreERT2*^ [#017593, *Sox2*^*tm1(cre/ERT2)Hoch*^]^13^ and *Atoh1-CreERT2* [#007684, Tg(Atoh1-cre/Esr1*)14Fsh]^41^ were obtained from The Jackson Laboratory. The *Hprt*^*TBX2*^ strain is available upon request from Andreas Kispert.

Embryos for *Tbx2*/TBX2 expression analyses were derived from matings of NMRI wild-type mice. For generation of loss-of-function mutants *Sox2*^*CreERT2/+*^*;Tbx2*^*fl/fl*^ males were mated with *Tbx2*^*fl/fl*^*;R26*^*mTmG/mTmG*^ females to obtain *Sox2*^*CreERT2/+*^*;Tbx2*^*fl/fl*^*;R26*^*mTmG/+*^ (*Sox2-Tbx2LOF*) mice, and *Atoh1-CreERT2/+;Tbx2*^*fl/fl*^ males with *Tbx2*^*fl/fl*^*;R26*^*mTmG/mTmG*^ females to obtain *Atoh1-CreERT2/+;Tbx2*^*fl/fl*^*;R26*^*mTmG/+*^ (*Atoh1-Tbx2LOF*) mice. For generation of misexpression mutants *Sox2*^*CreERT2/+*^ males were mated with *Hprt*^*TBX2/TBX2*^ females to obtain *Sox2*^*CreERT2/+*^*;Hprt*^*TBX2/Y(+)*^ (*Sox2-TBX2ME*) mice, and *Atoh1-CreERT2/+* males with *Hprt*^*TBX2/TBX2*^ females to obtain *Atoh1-CreERT2/+; Hprt*^*TBX2/Y(+)*^ (*Atoh1-TBX2ME*) mice. For generation of *mTmG*-positive controls, *Sox2*^*CreERT2/+*^ and *Atoh1-CreERT2/+* males were mated with *R26*^*mTmG/mTmG*^ females to obtain *Sox2*^*CreERT2/+*^*;R26*^*mTmG/+*^ and *Atoh1-CreERT2/+;R26*^*mTmG/+*^ mice, respectively. *Cre*-negative littermates or *Sox2*^*CreERT2/+*^*;R26*^*mTmG/+*^ and *Atoh1-CreERT2/+;R26*^*mTmG/+*^ mice served as controls.

For embryonic staging, the detection day of a vaginal plug was defined as embryonic day (E) 0.5. To induce recombination, 4 mg of tamoxifen (#T5648, Sigma-Aldrich) dissolved in corn oil (#C8267, Sigma-Aldrich) were orally applied to timed pregnant or breast-feeding dams in a single pulse at E12.5, E15.5, E16.5 or P0-1. From E15.5 onwards, 2 mg of progesterone (#P8783, Sigma-Aldrich) were additionally applied. Pregnant females and juvenile mice were euthanized by cervical dislocation, neonates were euthanized by decapitation. Embryos at E14.5, E16.5 and E18.5, neonates at P4 and juveniles at P17/21 were collected for analyses. Both sexes were interchangeably used with the exception of *Sox2-TBX2ME* mice, where we used male mice at embryonic stages and female mice at postnatal stages. The sample size was not predetermined by statistical methods. The experiments were not blinded due to the obvious phenotype. For all experiments, animals were assigned to groups based on their genotype.

### Tissue collection and preparation

Embryos were dissected in phosphate-buffered saline (PBS) and decapitated. For organ of Corti whole-mount preparations, cochleae were isolated in PBS and non-sensory epithelia (stria vascularis, Reissner’s membrane) were removed. Embryonic heads and cochlear whole-mounts were then fixed overnight in 4% paraformaldehyde (PFA)/PBS (#A3813, AppliChem) at 4 °C, dehydrated through an ascending methanol series (diluted in PBS) and stored in absolute methanol at −20 °C until use.

For collection of older tissue (P21), inner ears were isolated in PBS after decapitation, fixed in 4% PFA overnight and decalcified for 2 days in 0.5 M EDTA/PBS (#A2937, AppliChem), pH7.4, at 4 °C. Whole organs of Corti were isolated from inner ears after decalcification and dissected into three pieces (basal, medial, apical). Inner ears and cochlear whole-mounts were then dehydrated and stored in absolute methanol at −20 °C until use.

Genotypes were determined by sorting of GFP-expressing tissues and by PCR on genomic DNA prepared from embryonic tissues or ear clips.

### Histological analysis

Embryonic heads or postnatal inner ears were embedded in paraffin wax and sectioned to 5-µm. Sections were stained with Hematoxylin (#GHS332, Sigma-Aldrich) for 45 s, blued in 0.5% sodium-acetate for 1 min and counterstained with Eosin Y solution (#HT110132, Sigma-Aldrich) for 60-90 s.

### Immunohistochemistry on sections

Detailed information on antibodies, including order number and working concentrations, can be found in Supplementary Table 5. Tissue was embedded in paraffin wax and sectioned to 5-µm, followed by deparaffinization in Roti-Histol (#6640, Roth) and rehydration in a descending ethanol/H_2_O series. For embryonic sections (E14.5, E16.5, E18.5), epitope retrieval was accomplished with citrate-based antigen unmasking solution (#H-3300, Vector Laboratories) for 15 min at 100 °C. Postnatal tissue sections (P17/21) were subjected to heat-induced antigen retrieval 10 mM Tris-HCl/2 mM EDTA buffer, pH 9.0, for 90 min at 80 °C. Endogenous peroxidase activity was blocked by incubation in 3% H_2_O_2_/PBS (#AP121076.1211, AppliChem) for 15 min at room temperature (RT). All washing steps were performed using 0.1% Tween-20 in PBS (PBST). Samples were blocked with TNB Blocking Buffer (#FP1012, PerkinElmer) for 1 h. For antibodies generated in mice an additional IgG blocking step was performed using the M.O.M. Mouse Ig Blocking Reagent (#MKB-2213-1, Vector Laboratories). After blocking, sections were incubated with primary antibodies (diluted in TNB) overnight at 4 °C, followed by appropriate secondary and sometimes tertiary antibodies in TNB for 1 h at RT. Sections were counterstained with DAPI (0.1 µg/ml in PBST, #6335, Roth) for 10 min and mounted with Immunoselect Antifading Mounting Medium (#SCR-038447, Dianova).

For weakly expressed proteins, biotin-labelled secondary antibodies (1 h at RT) and the Tyramide Signal Amplification Kit (TSA, #NEL702001KT/NEL701001KT, PerkinElmer) were used for signal detection. HRP-conjugated streptavidin was diluted in TNB (1:100) and applied to sections for 30 min to 1 h at RT, whereas incubation with TSA reagent was performed for 10 to 15 min at RT in accordance with the manufacturer’s protocol.

For sequential double amplification, a 30 min 6% H_2_O_2_/PBS and an Avidin/Biotin block (15 min each, #SP-2001, Vector Laboratories) were performed in between the two amplification steps.

For sequential double labelling with two primary antibodies generated in the same host, masking of the first primary antibody was performed using the following unconjugated FAB fragments: goat-anti-rabbit FAB fragment (#111-007-003, Dianova), donkey-anti-mouse FAB fragment (#715-007-003, Dianova).

### Immunohistochemistry on cochlear whole-mount preparations

Cochlear whole-mounts were rehydrated in a descending methanol/PBS series. For the first permeabilization step, whole-mounts were treated with proteinase K (10 µg/ml, #RP102B, 7Bioscience) diluted in buffer for 2 min at RT. Digestion was stopped by washing in 0.2% glycine/PBS (#A1067, Applichem). For all following permeabilization or washing steps 0.3% Triton X-100 in PBS was used. Endogenous peroxidase activity was blocked by incubation in 3% H_2_O_2_/PBS for 15 min at RT. Samples were blocked with 20% heat-inactivated FCS in PBS-Triton for 1 h at RT, followed by an overnight incubation with primary antibodies, diluted in blocking solution, at 4 °C. Appropriate secondary and sometimes tertiary antibodies were applied to cochlear whole-mounts for 2 h at RT.

For detection of BCL11B, antigen retrieval was required prior to the blocking step. This was performed using 10 mM sodium citrate, pH 6 with 0.25% Triton X-100 for 30 min at 90 °C, followed by cooling down at RT for 30 min.

For visualization of NGFR (P75^NTR^), a biotin-labelled secondary antibody (2 h at RT) and the TSA Kit were used. Cochlear whole-mounts were incubated with the staining reagent for 30 min at RT, followed by washing with PBS-Triton on ice and inactivation of the horseradish peroxidase by incubation in 6% H_2_O_2_/PBS for 30 min at RT.

For co-staining of SLC17A8 and F-actin, non-methanol treated cochlear whole-mounts were used and counterstained with Phalloidin-iFluor488 (#ab176753, abcam, 1:1500) for 1 h at RT.

If required, cochlear whole-mount preparations were counterstained with DAPI. All specimens were mounted in Immunoselect Antifading Mounting Medium (SCR-038447, Dianova).

### Generation of antisense RNA in situ probes

In vitro transcription of digoxigenin-labelled-antisense RNA probes from DNA templates was performed using digoxigenin-labelling mix (#11277073910, Sigma) and T7/T3/SP6-RNA-Polymerases (#M0251/11031163001/M0207, NEB/Sigma-Aldrich).

For transcripts, for which no DNA template was available, primers pairs were designed using the MacVector software (version 16.0.8), modified with an additional T7-promoter sequence at the 5′ end of the reverse primer, and used for PCR based amplification of DNA templates from genomic DNA or cDNA. Accuracy of all probe templates was confirmed by sequencing.

Primer sequences used for amplification of DNA templates are listed in Supplementary Table 6.

### RNA in situ hybridization

In situ hybridization analysis was performed on 10-µm paraffin sections following a standard procedure with digoxigenin-labelled antisense riboprobes^45^. In brief, sections were deparaffinized, rehydrated in a descending ethanol/water series, and then treated with 10 µg/ml proteinase K (#RP102B, 7Bioscience) diluted in Tris-HCl buffer solution, pH 7.4, for 7 min at 37 °C. Followed by a 5 min incubation in 0.2% glycine/PBS, and re-fixation in 4% PFA/0.2% glutaraldehyde for 20 min at RT. Hybridization with digoxigenin-labelled riboprobes was performed overnight at 70 °C. After two 30 min washing steps in saline sodium citrate/formamide at 65 °C and a blocking step at RT, sections were incubated with sheep-anti-digoxigenin Fab fragments conjugated with alkaline phosphatase (1:4000, #11093274910, Roche) for 2 h at RT. Staining reaction was developed using the BM Purple AP substrate precipitating solution (#11442074001, Sigma Aldrich) to localize bound anti-digoxigenin antibody.

### Microarray analysis of cochlear ducts

For the E14.5 microarray analysis, whole cochlear ducts were isolated in Leibovitz’s L15 Medium (#F1315, Biochrom) and the spiral ganglion was removed. For the E18.5 microarray analysis, cochlear ducts were isolated in Leibovitz’s L15 Medium and dissected into two halves. The spiral ligament and the spiral ganglia were removed from the basal half and the remaining sensory tissue was collected for analysis. Immediately after dissection/isolation the tissue was frozen using dry ice and stored at −80 °C until use.

For the E14.5 microarray analysis, four independent sex-sorted pools per genotype, each containing 23–27 cochlear ducts, were collected from *Sox2-Tbx2LOF* mutants and cre-negative controls after a single pulse of tamoxifen at E12.5. For the E18.5 microarray analysis four independent sex-sorted pools per genotype, each containing 16–20 organs of Corti from the basal half of the cochlear duct, were collected from *Sox2-Tbx2LOF* mutants and cre-negative controls after a single pulse of tamoxifen at E16.5. After total RNA extraction using the peqGOLD RNAPure reagent (#732-3312, Peqlab) according to the manufacturer’s instructions, samples were sent to the Research Core Unit Transcriptomics of Hannover Medical School where they were hybridized to Agilent Whole Mouse Genome Oligo v2 (4x44K) Microarrays (#G4846A, Agilent Technologies) in a dual-colour mode.

Deregulated genes were determined with the software *Significance Analysis of Microarrays* 5.0 (SAM, Stanford University, CA, USA)^46^. For the analysis normalized expression data was assessed by two class unpaired comparison with *t*-statistics (*n* = 4). The value of *k* for the *k*-nearest-neighbor algorithm was set to 10 (default), the number of permutations was 100 (default). For the significance cut-off we used a minimum fold change of 1.5 and values for *delta* (1.39 for E14.5, 1.15 for E18.5) that resulted in an estimated false discovery rate (FDR) of zero.

### Image acquisition and analysis

Sections were photographed using the Leica DM5000 microscope with a Leica DFC300FX digital camera and Leica Firecam for Mac 1.9 software or the Leica DM6000 microscope with a Leica DFC350FX digital camera and Leica Application Suite X (3.0.1) software. Images of cochlear whole-mounts were acquired using the confocal laser scanning microscope Leica TCS SP8 and HC PL APO 20x/0.75 IMM, 40×/1.10 WATER, 63×/1.40 OIL CS2 objectives and Leica LAS X 3.5.5 software. Images were processed using Leica LAS X 3.0.1 (3D-reconstructions), FIJI/ImageJ 2.3.0 and Adobe Photoshop CS4.

### Quantifications and statistical analysis

Quantification of specific cell types on cochlear whole-mounts at E18.5 occurred in two steps. First, a 500 µm-long region of the organ of Corti at the mid-basal level of the cochlear duct was defined. Second, MYO6^+^ cells were counted within this area (approx. 260 cells per sample) and each and every hair cell was analysed for expression of BCL11B and/or SLC17A8. For analysis at P21, we defined 400-500 µm-long regions at the basal level of the organ of Corti and MYO6^+^ hair cells were analysed for expression of SLC25A6 and SLC17A8. Measurements and cell counts were performed using FIJI/ImageJ 2.3.0 (NIH) and Adobe Photoshop CS4 software. Cell counts were normalized to a length of 100 µm of cochlea in Microsoft Excel 16.16.27.

Statistical analyses were performed using GraphPad Prism7 and JASP (version 0.13.1). Normal distribution of data was tested using the D’Agostino & Pearson and Shapiro-Wilk normality test (normal distribution if *p*-values ≥ 0.05) and Q-Q-plots. Equality of variances was assessed by performing the F- and Levene’s test (equal variances if *p*-values ≥ 0.05). For comparison of two groups with normal distribution (parametric) and equal variances we used the unpaired two-tailed t-test. For comparison of two groups with normal distribution and unequal variances we used the two-tailed Welch’s t-test. For comparison of two groups with nonparametric distribution we used the two-tailed Mann-Whitney U test. Results were expressed as mean ± SD. A *p*-value < 0.05 was considered statistically significant.

For the quantification of type I afferent innervation of ectopic OHCs and ectopic IHCs in *A*t*oh1-Tbx2LOF* and *Atoh1-TBX2ME* cochleae, respectively, at P21, apical regions of the organ of Corti were stained for CALB2, which is strongly expressed in Type I afferents and IHCs and weakly in OHCs. Weak-CALB2-positive ectopic OHCs or strong-CALB2-positive ectopic IHCs were counted in single or multiple non-overlapping maximum-intensity-projection-images of the organ of Corti per individuum each covering a length of 246 µm (summing up to regions with lengths of 246 to 738 µm). Then the overall-percentage of these cells, that were innervated with CALB2-positive afferents, was determined. Due to low recombination in P21 *Atoh1-Tbx2ME* (Tamoxifen at E15.5) mutants, all CALB2^+^ ectopic IHCs in the entire apical region were counted.

For the quantification of the stereocilia phenotypes of ectopic OHCs and IHCs in *A*t*oh1-Tbx2LOF* and *Atoh1-TBX2ME* cochleae at P21, all SLC17A8-positive (ectopic) IHCs and SLC17A8-negative (ectopic) OHCs were analysed in single or multiple non-overlapping z-stacks of the organ of Corti per individuum each covering a length of 74 µm. We counted the cells of a given cell type according to their stereocilia phenotype and calculated the (weighted) percentages. For several mutants, OHC stereocilia were not included in the z-stacks, and therefore neglected for the cell counts.

### Reporting summary

Further information on research design is available in the Nature Portfolio Reporting Summary linked to this article.

## Supplementary information

Supplementary Information

Peer Review File

Reporting Summary

## Data Availability

All relevant data in this study are available within the article, Supplementary Data or Source Data. Microarray data have been deposited in Gene Expression Omnibus under the accession numbers GSE180500 and GSE180501. Source data are provided with this paper.

## Source data

Source Data
