# Supplementary material for: TBX2 specifies and maintains inner hair and supporting cell fate in the Organ of Corti
Source: Nat Commun. 2022 Dec 9;13:7628. doi: 10.1038/s41467-022-35214-4 (PMC9734556; doi:10.1038/s41467-022-35214-4)
Supplement: Supplementary file 1 — Supplementary Information [file 41467_2022_35214_MOESM1_ESM.pdf]

Supplementary Information

TBX2 specifies and maintains inner hair and supporting cell fate in the Organ of Corti

Marina Kaiser[1], Timo Lüdtke[1], Lena Deuper[1], Carsten Rudat[1], Vincent M. Christoffels[2], Andreas Kispert[1,*] & Mark-Oliver Trowe[1,*]

Supplementary Fig. 1

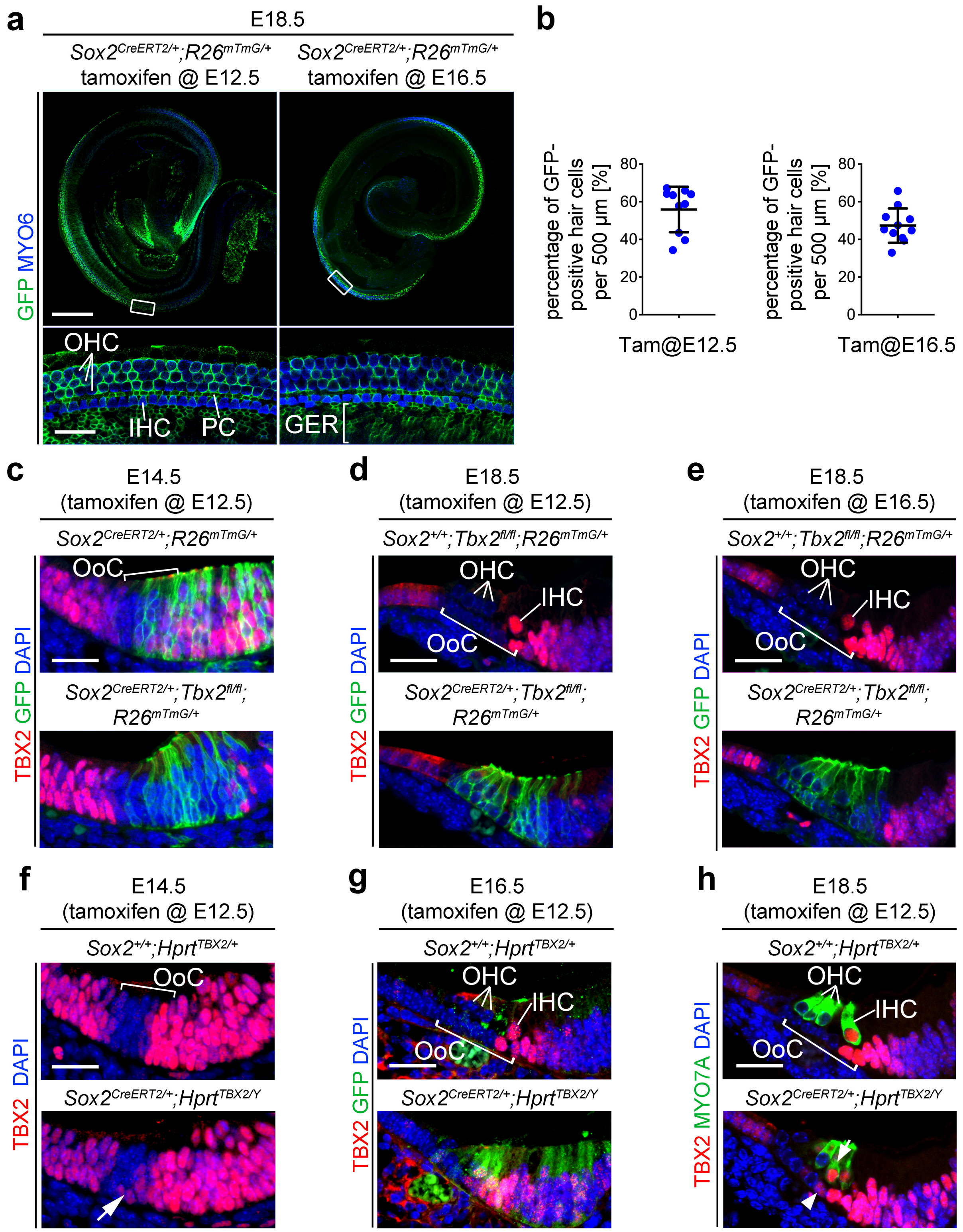

Supplementary Fig. 1 | Efficient manipulation of TBX2 expression in the prosensory region of the cochlea using the *Sox2^{CreERT2}* mouse line. **a-h** Immunofluorescence analyses of GFP expression from a *R26^{mTmG}* reporter and of TBX2 after tamoxifen-induced activation of the *Sox2^{CreERT2}* driver line at two different time points (E12.5, E16.5) in cochlear whole-mount preparations (**a,b**) and on cross-sections of the cochlea at the mid-basal level (**c-h**). **a,b** Analysis of GFP expression in E18.5 *Sox2$^{CreERT2/+}$;R26$^{mTmG/+}$* embryos after a single pulse of tamoxifen at E12.5 or E16.5 shows that the *Sox2^{CreERT2}* line leads to efficient recombination in MYO6^{+} hair cells, pillar cells (PCs) and cells of the adjacent greater epithelial ridge (GER) (**a**). **b** Quantification of GFP^{+} hair cells (MYO6^{+}) in the organ of Corti. n=10 (tamoxifen administration at E12.5), n=11 (tamoxifen administration at E16.5). Mean±standard deviation. Source data are provided as a Source Data file. **c,d,** Analysis of TBX2 expression in *Sox2$^{CreERT2/+}$;Tbx2$^{fl/fl}$;R26$^{mTmG/+}$* and control (*Sox2$^{CreERT2/+}$;R26$^{mTmG/+}$* or *Sox2$^{+/+}$;Tbx2$^{fl/fl}$;R26$^{mTmG/+}$*) cochleae at E14.5 (**c**) and E18.5 (**d**) after a single pulse of tamoxifen at E12.5. Expression of TBX2 is completely lost in recombined (GFP^{+}) cells of the developing organ of Corti (OoC) and in cells of the adjacent GER. n=3 for each genotype. **e** Analysis of TBX2 expression in *Sox2$^{CreERT2/+}$;Tbx2$^{fl/fl}$;R26$^{mTmG/+}$* and control (*Sox2$^{+/+}$;Tbx2$^{fl/fl}$;R26$^{mTmG/+}$*) cochleae at E18.5 after a single pulse of tamoxifen at E16.5. Expression of TBX2 is completely lost in recombined (GFP^{+}) hair and supporting cells of the organ of Corti. n=3 for each genotype. **f-h** Analysis of TBX2 expression at E14.5 (**f**), E16.5 (**g**) and E18.5 (**h**) and of GFP expression at E16.5 (**g**) in *Sox2$^{CreERT2/+}$;Hprt$^{TBX2/Y}$* and control (*Sox2$^{+/+}$;Hprt$^{TBX2/+}$*) cochleae after a single pulse of tamoxifen at E12.5. At E14.5, TBX2^{+} cells can be observed in the outer compartment of the organ of Corti (arrow in **f**). Arrow and arrowhead in **h** point to hair and supporting cells with ectopic TBX2 expression, respectively. n=3 for each genotype. Nuclei were counterstained with DAPI. Scale bars: 250 µm (overview in **a**), 30 µm (**c-h**).

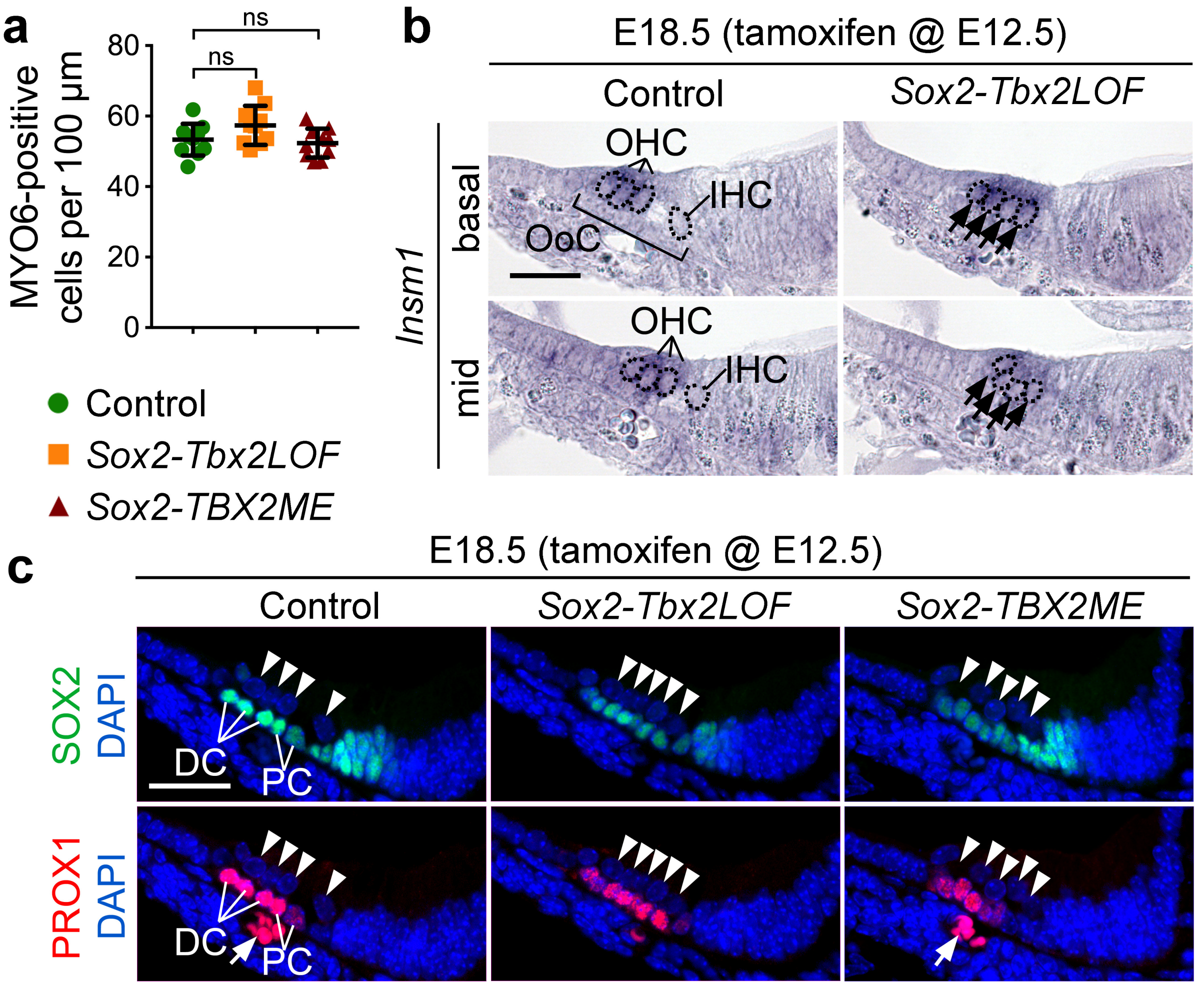

Supplementary Fig. 2 | Increased number of OHCs at the expense of IHCs upon inactivation of *Tbx2* in prosensory cells at E12.5. a-c Analysis of hair and supporting cell differentiation in mice in which *Tbx2* was ablated (*Sox2-Tbx2LOF*) or misexpressed (*Sox2-TBX2ME*) in prosensory cells by tamoxifen administration at E12.5. **a** Quantification of the total number of hair cells ($MYO6^{+}$) at the mid-basal level of cochlear whole-mount preparations of E18.5 *Sox2-Tbx2LOF*, *Sox2-TBX2ME* and control embryos; n=10 for each genotype. Mean±standard deviation, two-sided unpaired t-test. ns, not significant. Exact p-values and related source data are provided as a Source Data file. **b** RNA *in situ* hybridization analysis of *Insm1* expression in E18.5 control and *Sox2-Tbx2LOF* cochleae. In the control, *Insm1* is weakly expressed in the three OHCs but not in IHCs at the basal as well as medial (mid) level of the cochlear duct. Upon *Tbx2* inactivation, the number of *Insm1*-expressing cells increased (arrows). n=3 for each genotype. Dotted circles mark the nuclei of hair cells in the organ of Corti (OoC). **c** Single channels of PROX1 and SOX2 immunofluorescence analysis at E18.5 (see also Fig. 2c). n=6 for each genotype. Arrowheads point to the nuclei of hair cells, arrows to erythrocytes. DC, Deiters' cells; PC, pillar cells. Scale bars: 30 µm.

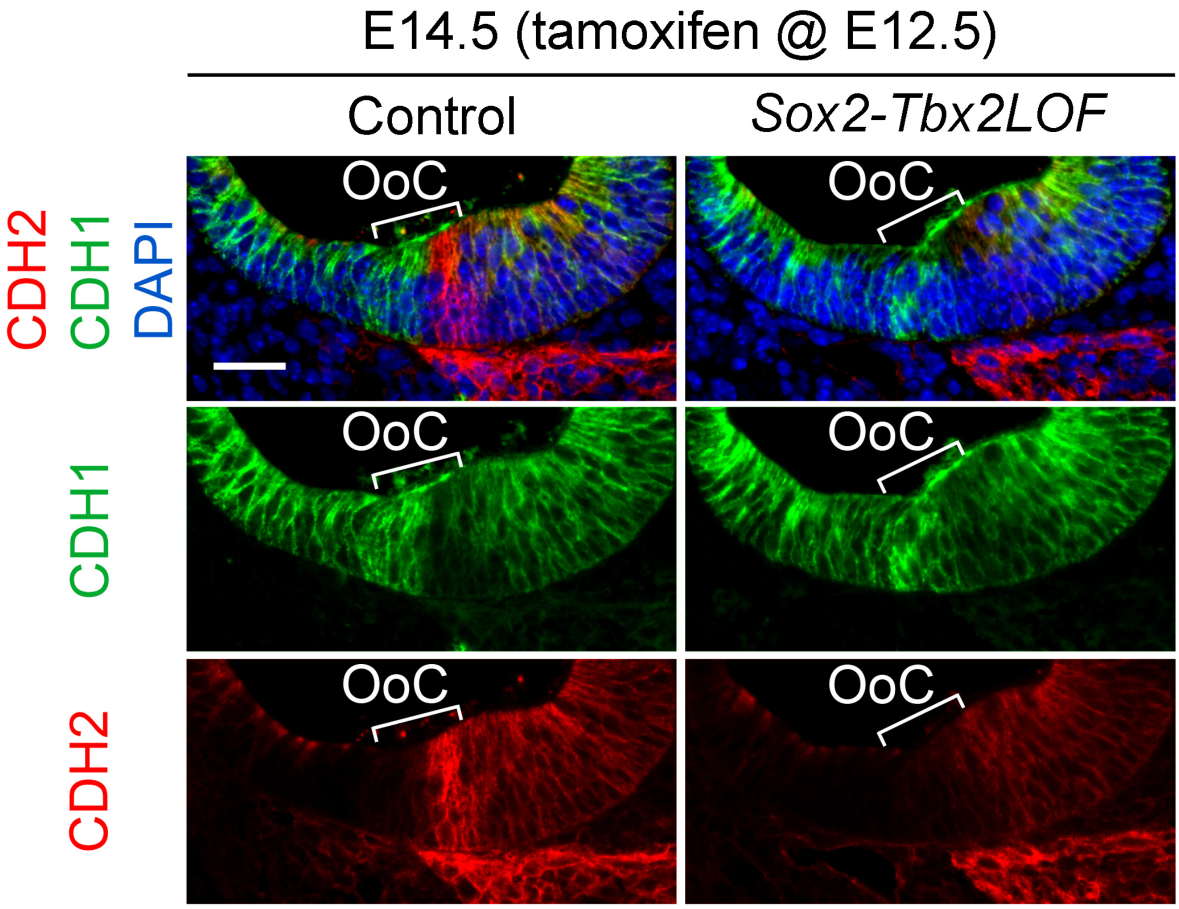

Supplementary Fig. 3 | The CDH2⁺ inner compartment of the organ of Corti is not established upon inactivation of *Tbx2* in prosensory cells at E12.5. Immunofluorescence analysis of CDH1 and CDH2 expression in the developing organ of Corti (OoC) of E14.5 *Sox2-Tbx2LOF* and control embryos after administration of tamoxifen at E12.5. n=3 for each genotype. The strong CDH2 expression, which marks the inner compartment of the developing OoC, is absent in *Tbx2*-deficient cochleae. Nuclei are counterstained with DAPI. Scale bars: 30 µm.

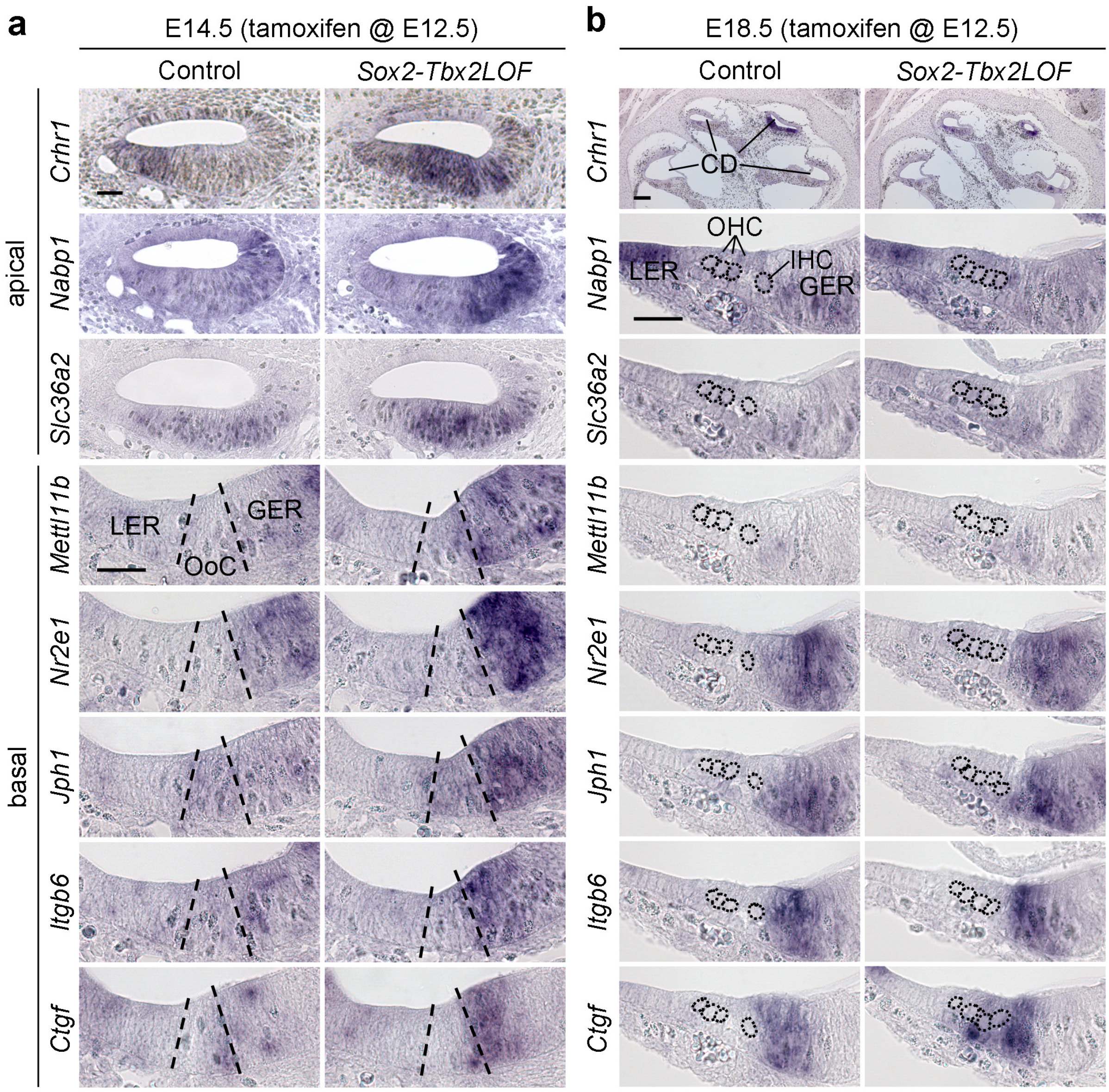

Supplementary Fig. 4 | The patterning of the cochlear epithelium is affected upon early *Tbx2* inactivation in prosensory cells. a,b RNA *in situ* hybridization analysis on cochlear sections of transcripts significantly upregulated in a microarray analysis of E14.5 *Sox2-Tbx2LOF* cochlear ducts after administration of tamoxifen at E12.5. Spatial distribution is shown at E14.5 (**a**) and E18.5 (**b**). Expression of *Crhr1*, *Nabp1* and *Slc36a2* is upregulated in the most apical turn of *Tbx2*-deficient cochleae at E14.5, whereas *Mettl11b*, *Nr2e1*, *Jph1*, *Itgb6* and *Ctgf* are upregulated or ectopically expressed in the greater epithelial ridge (GER) in the basal turn. At E18.5, *Crhr1* expression is restricted to the apical turn and *Nabp1* is expressed in cells of the outer sulcus in control and *Sox2-Tbx2LOF* embryos. *Slc36a2* and *Mettl11b* are not expressed at E18.5, whereas *Nr2e1*, *Jph1*, *Itgb6* and *Ctgf* are still expressed in the GER. Dashed lines in (**a**) mark the outer borders of the developing organ of Corti (OoC). Dotted circles in (**b**) mark the nuclei of hair cells. n=3 samples for each genotype. Scale bars: 100 µm (for *Crhr1* in **b**), 30 µm. CD, cochlear duct; LER, lesser epithelial ridge.

Supplementary Fig. 5

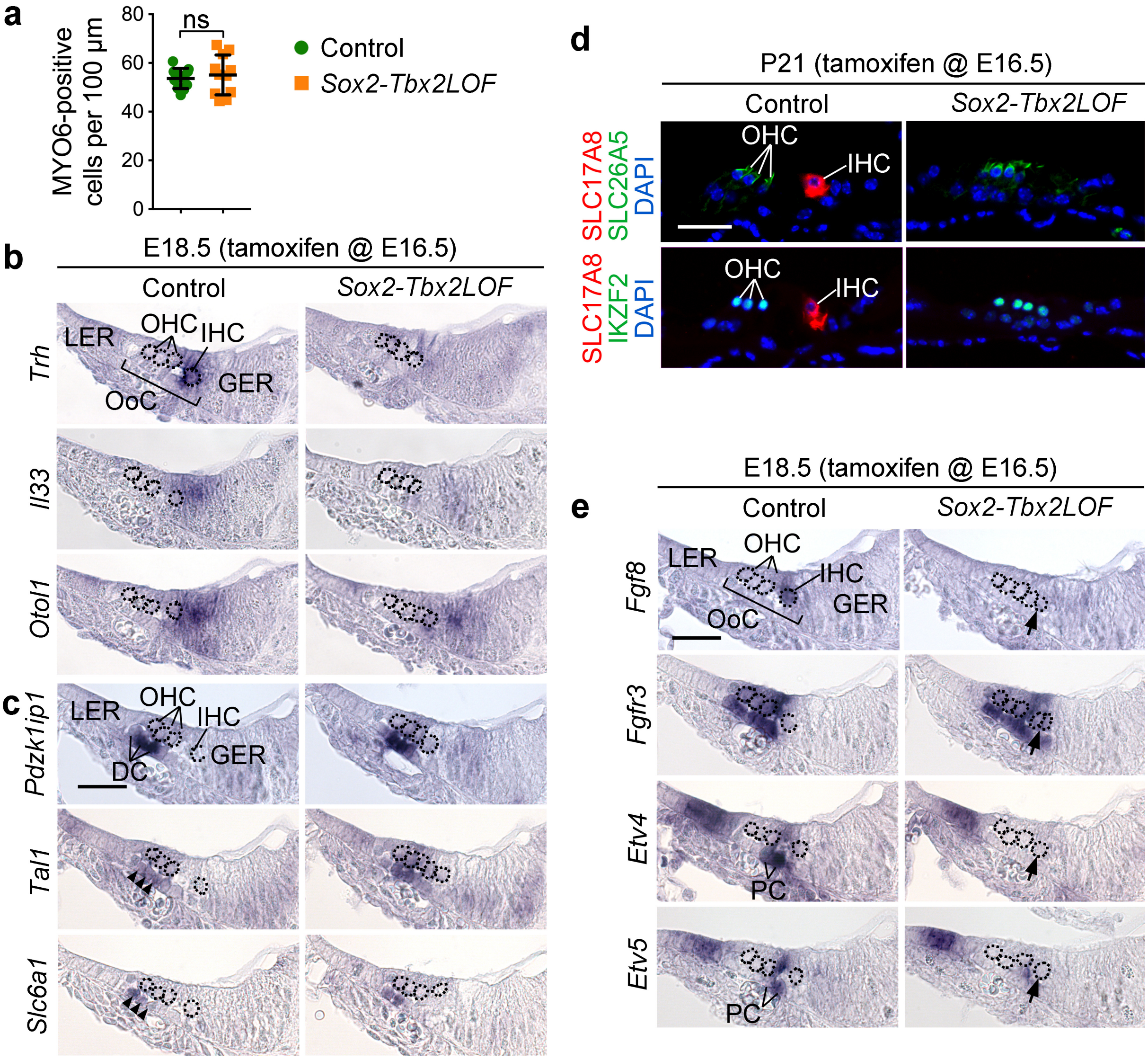

Supplementary Fig. 5 | Increased number of OHCs and a complete loss of IHCs upon late inactivation of *Tbx2 in* SOX2-positive cochlear cells. a-e Analysis of gene expression and hair cell differentiation in mice in which *Tbx2* was ablated (*Sox2-Tbx2LOF*) in $Sox2^{+}$ cells of the organ of Corti by tamoxifen administration at E16.5. **a** Quantification of the total number of hair cells ($MYO6^{+}$) at the mid-basal level of whole-mount preparations of E18.5 *Sox2-Tbx2LOF* and control organs of Corti. n=11 for each genotype. Mean±standard deviation, two-sided unpaired t-test with Welch's correction. ns, not significant. Exact p-values and related source data are provided as a Source Data file. **b,c** RNA *in situ* hybridization analysis of transcripts significantly down- (**b**) or up-regulated (**c**) in E18.5 *Sox2-Tbx2LOF* cochlear ducts. n=4 for each genotype. **b** *Trh* is expressed in inner hair and supporting cells, *Il33* and *Otol* in the inner compartment of the organ of Corti and in the greater epithelial ridge (GER). All 3 genes are downregulated in *Sox2-Tbx2LOF* cochleae. **c** *Pdzk1ip1*, *Tal1* and *Slc6a1* are expressed in a subset of Deiters' cells (DCs, arrowheads) in control and *Sox2-Tbx2LOF* cochleae. **d** Immunofluorescence analysis of expression of SLC17A8 (marks IHCs), SLC26A5 and IKZF2 (mark OHCs) on cross sections of P21 *Sox2-Tbx2LOF* cochleae compared to controls. Nuclei are counterstained with DAPI. n=5 for each genotype. **e** RNA *in situ* hybridization analysis of FGF signaling components in *Sox2-Tbx2LOF* cochleae at E18.5. Expression of *Fgf8* in the innermost hair cell and that of *Etv4* and *Etv5* in pillar cells (PCs) is lost or strongly downregulated upon late *Tbx2*-inactivation. Expression of *Fgfr3*, which marks DCs and PCs, is expanded medially in *Sox2-Tbx2LOF* cochleae n=4 for each genotype. Arrow marks the innermost hair cell. Dotted circles (**b,c,e**) outline the nuclei of hair cells. Scale bars: 30 µm. GER, greater epithelial ridge; LER, lesser epithelial ridge; OoC, organ of Corti.

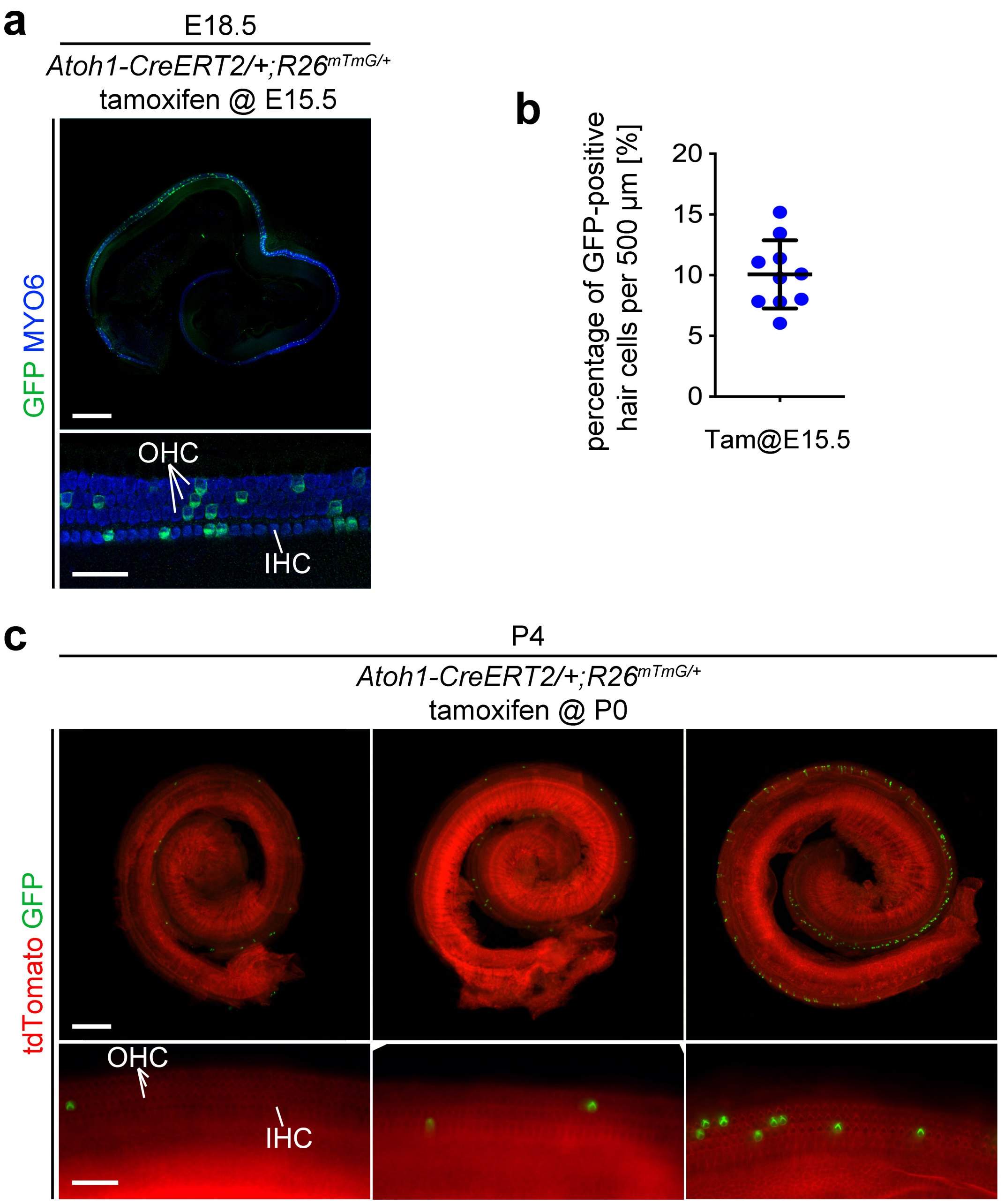

Supplementary Fig. 6 | The *Atoh1-CreERT2* mouse line allows hair cell-specific manipulation of TBX2 expression. a,**b** Immunofluorescence analysis of GFP expression in cochlear whole-mount preparations of E18.5 *Atoh1-CreERT2/+;R26$^{mTmG/+}$* embryos subjected to administration of tamoxifen at E15.5 (**a**). The *Atoh1-CreERT2* line induces recombination of the *R26^{mTmG}* reporter allele in approximately 10% of hair cells (MYO6^{+}) (**b**). n=5 biologically independent samples**.** Mean±standard deviation. Source data are provided as a Source Data file. **c** Epifluorescence analysis of native GFP and tdTomato expression in whole *Atoh1-CreERT2/+;R26$^{mTmG/+}$* cochleae at P4 after oral administration of tamoxifen to the feeding dam one day after birth (P0). Recombination (GFP^{+} cells) occurs in a variable numbers of hair cells in an apical-to-basal gradient. n=10 biologically independent samples. Scale bars: 250 µm (overviews in **a** and **c**), 30 µm (higher magnifications in **a** and **c**).

Supplementary Fig. 7

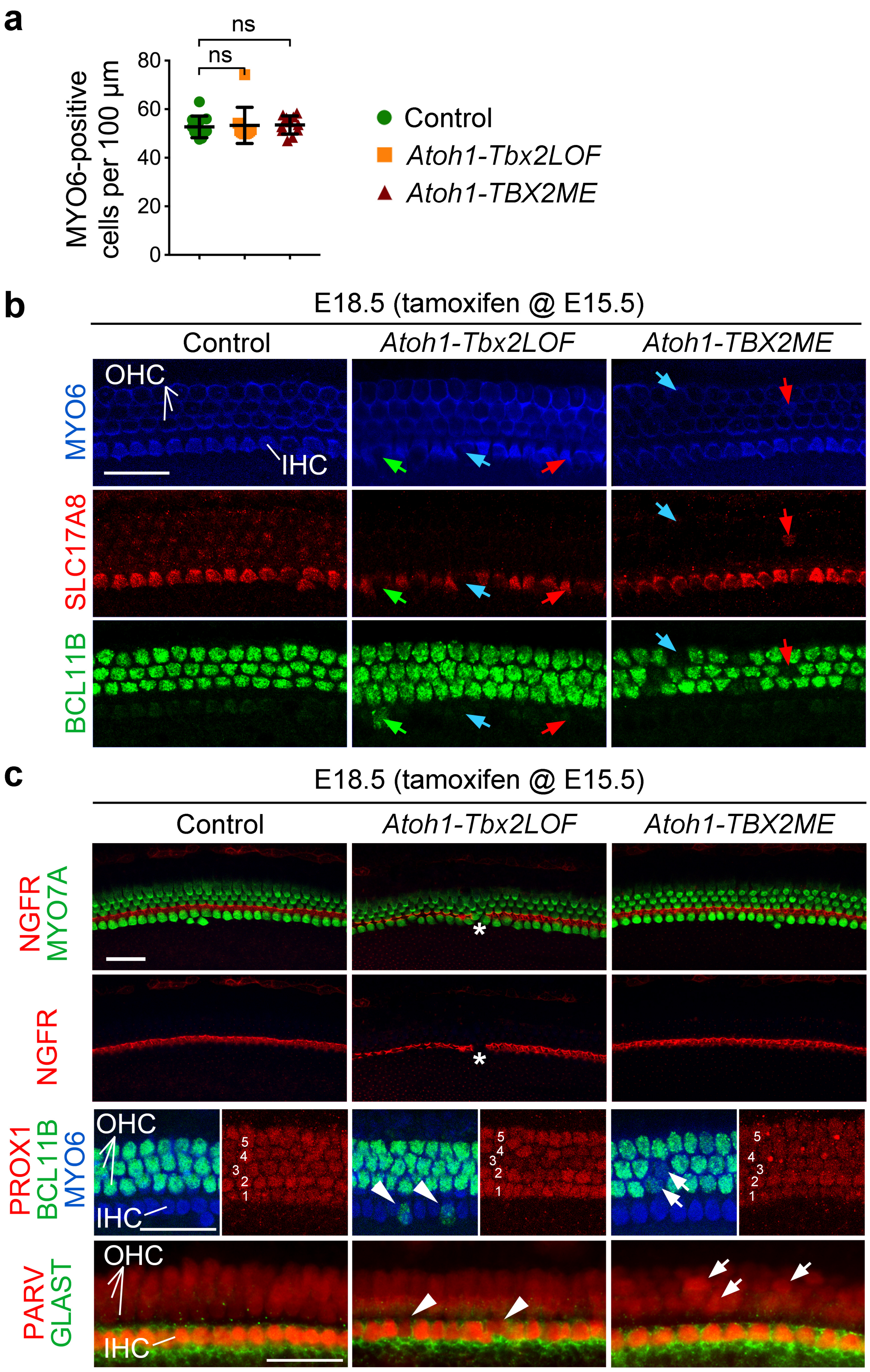

Supplementary Fig. 7 | Hair cell-specific manipulation of *Tbx2* expression leads to fate shifts in hair cells but not in underlying supporting cells at E18.5. a-c Analysis of hair and supporting cell differentiation in E18.5 embryos in which *Tbx2* was ablated (*Atoh1-Tbx2LOF*) or misexpressed (*Atoh1-TBX2ME*) in individual hair cells by tamoxifen administration at E15.5. **a**, Quantification of the total number of hair cells ($MYO6^{+}$) at the mid-basal level of whole-mount preparations of E18.5 control (n=10), *Atoh1-Tbx2LOF* (n=10), *Atoh1-TBX2ME* (n=11) cochleae. Mean±standard deviation, two-sided Mann-Whitney or unpaired t-test. ns, not significant. Exact p-values and related source data are provided as a Source Data file. **b** Single color channels of immunofluorescence for MYO6 (all hair cells), SLC17A (IHCs) and BCL11B (OHCs); for merged images see Fig. 5a. Red arrow points to $MYO6^{+}/SLC17A8^{+}$, green arrow to a $MYO6^{+}/BCL11B^{+}$ and blue arrow to a double-negative ($SLC17A8^{-}/BCL11B^{-}$) hair cell. **c** Co-immunofluorescence of supporting and hair cell markers at the mid-basal to mid-level of whole-mount preparations of E18.5 organs of Corti of control (n=8), *Atoh1-TBX2LOF* (n=6) and *Atoh1-TBX2ME* (n=7) mutants. Markers are: NGFR (pillar cells), PROX1 (Deiters' and pillar cells), GLAST (inner phalangeal cells and inner border cells), MYO6/MYO7A (all hair cells), BCL11B (OHCs), PARV (strong in IHCs, weak in OHCs). Asterisk marks a gap in the row of $NGFR^{+}$ pillar cells in the *Atoh1-TBX2LOF* organ of Corti. Note that the cellular pattern of hair cells is mildly disrupted with some hair cells being located in the pillar cell region. For the PROX1/BCL11B co-staining two different focus planes are shown and rows of $PROX1^{+}$ supporting cells are numbered (row 1 and 2: pillar cells, rows 3-5: OSCs). Arrowheads point to induced OHC-like cells in the inner compartment of the organ of Corti. Arrows point to ectopic IHC-like cells in the outer domain of the organ of Corti. Scale bars: 30 µm.

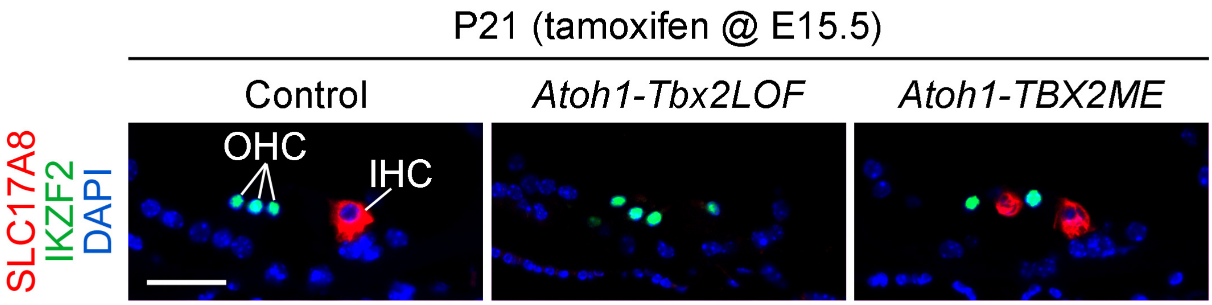

Supplementary Fig. 8 | Manipulation of *Tbx2* expression in hair cells leads to fate shifts at postnatal stages. Analysis of hair cell differentiation in P21 mice in which *Tbx2* was ablated (*Atoh1-Tbx2LOF*) or misexpressed (*Atoh1-TBX2ME*) in individual hair cells by tamoxifen administration at E15.5. Immunofluorescence analysis of SLC17A8 (marks IHCs) and IKZF2 (mark OHCs) expression on cochlear cross sections of P21 control (n=6), *Atoh1-Tbx2LOF* (n=4) and *Atoh1-TBX2ME* (n=7) mice. Nuclei are counterstained with DAPI.

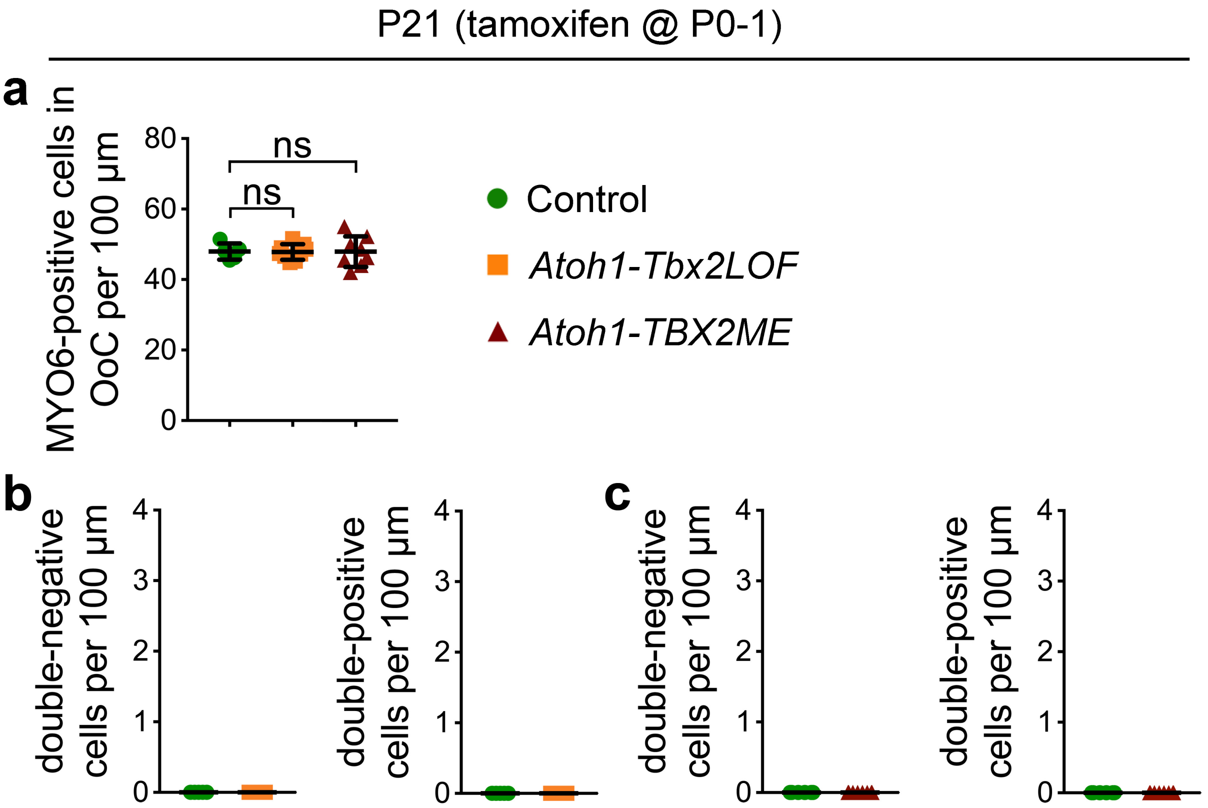

Supplementary Fig. 9 | Postnatal manipulation of *Tbx2* expression in hair cells leads to their complete transdifferentiation. Analysis of hair cell differentiation in P21 mice in which *Tbx2* was ablated (*Atoh1-Tbx2LOF*) or misexpressed (*Atoh1-TBX2ME*) in individual hair cells by tamoxifen administration at P0-1. **a-c** Quantification of the total number of hair cells ($MYO6^{+}$) (**a**) and of SLC26A5/SLC17A8 double-negative and double-positive hair cells in the inner compartment of P21 control (n=5) and *Atoh1-Tbx2LOF* organ of Corti (n=9) (**b**) and the outer compartment of control (n=6) and *Atoh1-TBX2ME* organ of Corti (n=8) (**c**) at the basal level. ns, not significant. Mean±standard deviation, two-sided unpaired t-test or Mann-Whitney-U test. Scale bar: 30 µm. Exact p-values and related source data are provided as a Source Data file.

Supplementary Table 1 | List of genes with significant downregulation in cochlear ducts of E14.5 *Sox2*$^{CreERT2/+}$*;Tbx2*$^{fl/fl}$ embryos after a single pulse of tamoxifen at E12.5. 4 different pools of control and mutant material were used for the microarray analysis. For genes marked by an asterisk mRNA or protein expression data are available in the literature. E, embryonic day; P, postnatal day.

Gene Symbol	FC	Literature	Reference
Stmn3	-6,1	expressed by embryonic and postnatal hair cells and in spiral ganglion neurons	Li et al., 2020 (PMID: 31913118, DOI: 10.7554/eLife.50491);<br>Scheffer et al., 2015 (PMID: 25904789, DOI: 10.1523/JNEUROSCI.5126-14.2015)
Foxa2	-4,2	x	
Trhr	-4,1	enriched in *Lfng*-GFP$^+$ cells at P1	Maass et al., 2016 (PMID: 27918591, DOI: 10.1371/journal.pone.0167286)
*Pvalb**	-4,1	expressed in inner hair cells at P8	Simmons et al., 2010 (PMID: 20653034, DOI: 10.1002/cne.22424)
Stmn4	-4,0	expressed in spiral ganglion neurons	Li et al., 2020 (PMID: 31913118, DOI: 10.7554/eLife.50491)
*Celf3**	-3,7	expressed in the stato-acoustic ganglion at E10.5	Kaiser et al., 2021 (PMID: 33795231, DOI: 10.1242/dev.195651)
Ptgdr	-3,4	x	
Foxd3	-3,0	expressed by supporting cells	Scheffer et al., 2015 (PMID: 25904789, DOI: 10.1523/JNEUROSCI.5126-14.2015)
Scrt1	-2,9	expressed in spiral ganglion neurons	Li et al., 2020 (PMID: 31913118, DOI: 10.7554/eLife.50491)
Prss35	-2,8	differentially expressed along the tonotopic axis of the cochlea at P8	Son et al., 2012 (PMID: 22808246, DOI: 10.1371/journal.pone.0040735)
*Pkd2l1**	-2,8	expressed in air cells, enriched in *Lfng-GFP*$^+$ cells at P1	Maass et al., 2016 (PMID: 27918591, DOI: 10.1371/journal.pone.0167286);<br>Wu et al., 2016 (PMID: 27196058, DOI: 10.1371/journal.pone.0155577)
*Grxcr1**	-2,8	expressed in stereocilia bundles of hair cells at P1 (higher levels in outer hair cells)	Odeh et al., 2010 (PMID: 20137774, DOI: 10.1016/j.ajhg.2010.01.016)
B3galt2	-2,7	x	
Vgf	-2,6	x	
Tmprss4	-2,4	x	
*Fgf8**	-2,4	expressed in inner hair cells from E16.5 on	Jacques et al., 2007 (PMID: 17634195, DOI: 10.1242/dev.02874)
Degs2	-2,3	x	
Trh	-2,3		
*Gad1**	-2,3	expressed in the greater epithelial ridge at P3-P7	Kim et al., 2016 (PMID: 26178595, DOI: 10.1002/dneu.22326)
*Smpx**	-2,3	expressed in hair cells at P1, mutation of *Smpx* results in hearing loss	Huebner et al., 2011 (PMID: 21549336, DOI: 10.1016/j.ajhg.2011.04.007);<br>Yoon et al., 2011 (PMID: 21519551, DOI: 10.5115/acb.2011.44.1.69)
Chl1	-2,2	expressed by supporting cells	Scheffer et al., 2015 (PMID: 25904789, DOI: 10.1523/JNEUROSCI.5126-14.2015)
Fbp2	-2,2	expressed in hair cells (*Pou4f3-eGFP*)	Hickox et al., 2017 (PMID: 28039372, DOI: 10.1523/JNEUROSCI.2267-16.2016)
Stk32a	-2,2	x	
Cdh20	-2,2	x	
Lrrtm1	-2,2	expressed by supporting cells	Scheffer et al., 2015 (PMID: 25904789, DOI: 10.1523/JNEUROSCI.5126-14.2015)

Gene Symbol	FC	Literature	Reference
Cdh19	-2,1	expressed by supporting cells	Scheffer et al., 2015 (PMID: 25904789, DOI: 10.1523/JNEUROSCI.5126-14.2015)
Foxa1	-2,1	x	
Chgb	-2,1	expressed by embryonic and postnatal hair cells	Scheffer et al., 2015 (PMID: 25904789, DOI: 10.1523/JNEUROSCI.5126-14.2015)
Pex5l	-2,1	expressed in hair cells (*Pou4f3-eGFP*)	Hickox et al., 2017 (PMID: 28039372, DOI: 10.1523/JNEUROSCI.2267-16.2016)
Gpr17	-2,1	x	
C030013G03Rik	-2,0	x	
*Fabp7**	-2,0	expressed by supporting cells, expressed in inner phalangeal cells (inner supporting cells)	Basch et al., 2016 (PMID: 27966429, DOI: 10.7554/eLife.19921); Scheffer et al., 2015 (PMID: 25904789, DOI: 10.1523/JNEUROSCI.5126-14.2015)
*Pou4f1**	-2,0	expressed in the medial compartment at E14.5?	Scherrill et al., 2019 (PMID: 31085606, DOI: 10.1523/JNEUROSCI.2728-18.2019)
Dhh	-2,0	x	
Chga	-1,9	expressed by embryonic and postnatal hair cells	Scheffer et al., 2015 (PMID: 25904789, DOI: 10.1523/JNEUROSCI.5126-14.2015)
Clvs1	-1,9	expressed in P4 organs of Corti	Lewis et al., 2016 (PMID: 26988146, DOI: 10.1038/srep23363)
Cadps	-1,9	x	
Cybrd1	-1,9	differentially expressed along the tonotopic axis of the cochlea at P8	Son et al., 2012 (PMID: 22808246, DOI: 10.1371/journal.pone.0040735)
Celsr3	-1,9	x	
Snap91	-1,8	expressed by embryonic and postnatal hair cells	Reisinger et al., 2010 (PMID: 20648058, DOI: 10.1038/ejhg.2010.111); Scheffer et al., 2015 (PMID: 25904789, DOI: 10.1523/JNEUROSCI.5126-14.2015)
Mall	-1,8	x	
E230025N22Rik	-1,8	enriched in apical spiral ganglion neurons, expressed by postnatal hair cells	Scheffer et al., 2015 (PMID: 25904789, DOI: 10.1523/JNEUROSCI.5126-14.2015); Shrestha et al., 2018 (PMID: 30078709, DOI: 10.1016/j.cell.2018.07.007)
*Olig1**	-1,8	*Olig1* expression overlaps a *Sox2*- and *Jagged1*-positive region at early stages	Kanaya et al., 2015 (PMID: 25778822, DOI: 10.1016/j.gep.2015.03.001)
Uts2b	-1,8	x	
Lppr5	-1,8	x	
Pdzph1	-1,8	x	
Fam150b	-1,7	x	
*Npy**	-1,7	expressed in inner pillar cells at P1	Kolla et al., 2020 (PMID: 32404924, DOI: 10.1038/s41467-020-16113-y)
Lor	-1,7	x	
Tmem28	-1,7	x	
Slc22a3	-1,7	expressed in supporting cells and downregulated upon DAPT-treatment	Campbell et al., 2016 (PMID: 26786414, DOI: 10.1038/srep19484)
Sis	-1,7	x	

Gene Symbol	FC	Literature	Reference
Mmd2	-1,7	enriched in vestibular ganglion at E12, downregulated upon DAPT treatment	Campbell et al., 2016 (PMID: 26786414, DOI: 10.1038/srep19484); Lu et al., 2011 (PMID: 21795542, DOI: 10.1523/JNEUROSCI.2358-11.2011)
Nefm	-1,7	expressed by embryonic and postnatal hair cells	Scheffer et al., 2015 (PMID: 25904789, DOI: 10.1523/JNEUROSCI.5126-14.2015)
*Otoa**	-1,7	expressed in cells immediately adjacent and medial to inner hair cells	Zwaenepoel et al., 2002 (PMID: 11972037, DOI: 10.1073/pnas.082515999)
*Nrcam**	-1,7	expressed in the greater epithelial ridge at E14 and restricted to the organ of Corti from E17.5	Brand et al., 2015 (PMID: 25407819, DOI: 10.1007/s12031-014-0436-y); Harley et al., 2018 (PMID: 29536590, DOI: 10.1002/dvdy.24629)
Ackr4	-1,7	x	
*Dlx1**	-1,7	expressed in chicken stato-acoustic ganglion	Brown et al., 2005 (PMID: 15672396, DOI: 10.1002/cne.20418)
*Ngfr**	-1,7	expressed in pillar cells from E17	Bartheld et al., 1991 (PMID: 1664321)
Npr3	-1,7	expressed by supporting cells	Scheffer et al., 2015 (PMID: 25904789, DOI: 10.1523/JNEUROSCI.5126-14.2015)
Cdk15	-1,7	x	
Lsamp	-1,7	expressed in the lateral prosensory domain, supporting cell-specific gene positively regulated by Notch	Chrysostomou et al., 2020 (PMID: 33127852, DOI: 10.1523/JNEUROSCI.1192-20.2020); Kolla et al., 2020 (PMID: 32404924, DOI: 10.1038/s41467-020-16113-y)
Cd1d2	-1,7	x	
Thbs4	-1,7	knockout mice (TSP4) do not show an auditory phenotype	Mendus et al., 2014 (PMID: 24460873, DOI: 10.1111/ejn.12486)
*Slitrk6**	-1,7	expressed in all upporting cells of the rgan of Corti between E15.5 and P1	Katayama et al., 2009 (PMID: 19936227, DOI: 10.1371/journal.pone.0007786); Maass et al., 2016 (PMID: 27918591, DOI: 10.1371/journal.pone.0167286)
NAP001637-001	-1,6	x	
Me1	-1,6	expressed by embryonic and postnatal hair cells	Scheffer et al., 2015 (PMID: 25904789, DOI: 10.1523/JNEUROSCI.5126-14.2015)
Dhrs2	-1,6	expressed by supporting cells	Scheffer et al., 2015 (PMID: 25904789, DOI: 10.1523/JNEUROSCI.5126-14.2015)
Prkcq	-1,6	differentially expressed along the tonotopic axis of the cochlea at P8	Son et al., 2012 (PMID: 22808246, DOI: 10.1371/journal.pone.0040735)
Gpr37	-1,6	x	
Pnmal1	-1,6	x	
Adgrg6	-1,6	x	
*Kcnma1**	-1,6	weakly expressed in adult inner hair cells	Lingle et al., 2019 (DOI: 10.1073/pnas.1907065116)
*Mreg**	-1,6	expressed in hair cells at P1	Cai et al., 2015 (PMID: 25855195, DOI: 10.1523/JNEUROSCI.5083-14.2015); Scheffer et al., 2015 (PMID: 25904789, DOI: 10.1523/JNEUROSCI.5126-14.2015)
LOC545466	-1,6	x	
Hpdl	-1,6	x	
Lhfpl4	-1,6	x	
Slc17a6	-1,6	enriched in spiral ganglion neurons	Li et al., 2020 (PMID: 31913118, DOI: 10.7554/eLife.50491)
Slc18a2	-1,6	x	
Ism1	-1,5	expressed by supporting cells	Scheffer et al., 2015 (PMID: 25904789, DOI: 10.1523/JNEUROSCI.5126-14.2015)

Gene Symbol	FC	Literature	Reference
Foxn4	-1,5	x	
Edn1	-1,5	mutations in Edn1 are associated with hearing impairment	Uchida et al., 2009 (PMID: 19358249, DOI: 10.1002/lary.20181)
Scn4b	-1,5	expressed in spiral ganglion neurons	Li et al., 2020 (PMID: 31913118, DOI: 10.7554/eLife.50491);<br>Shrestha et al., 2018 (PMID: 30078709, DOI: 10.1016/j.cell.2018.07.007)
Hopx	-1,5	expressed by embryonic and postnatal hair cells	Scheffer et al., 2015 (PMID: 25904789, DOI: 10.1523/JNEUROSCI.5126-14.2015)
Ednrb	-1,5	expressed in stira vascularis (melanocystes)	Renauld et al., 2021 (PMID: 33484097, DOI: 10.1111/pcmr.12961)
Frmd3	-1,5	differentially expressed along the tonotopic axis of the cochlea at P8	Son et al., 2012 (PMID: 22808246, DOI: 10.1371/journal.pone.0040735)
Gna14	-1,5	x	
*Lfng**	-1,5	expressed in the medial compartment around E14.5, expressed in all supporting cells at P1	Basch et al., 2016 (PMID: 27966429, DOI: 10.7554/eLife.19921)
Kndc1	-1,5	expressed by cochlear hair cells	Scheffer et al., 2015 (PMID: 25904789, DOI: 10.1523/JNEUROSCI.5126-14.2015)
Pde4dip	-1,5	expressed in hair cells	Hickox et al., 2017 (PMID: 28039372, DOI: 10.1523/JNEUROSCI.2267-16.2016)

Supplementary Table 2 | List of genes with significant upregulation in cochlear ducts of E14.5 *Sox2*$^{CreERT2/+}$*;Tbx2*$^{fl/fl}$ mutants after a single pulse of tamoxifen at E12.5. 4 different pools of control and mutant material were used for the microarray analysis. For genes marked by an asterisk mRNA or protein expression data are available in the literature. E, embryonic day; P, postnatal day.

Gene Symbol	FC	Literature	Reference
*Esr1**	10,2	expressed in inner and outer hair cells in 3-month old mice	Simonoska et al., 2009 (PMID: 19293293, DOI: 10.1677/JOE-09-0060)
Umodl1	3,3	expressed in *Atoh1-GFP+* cells at P1, enriched in P6 *Lfng-GFP*$^{+}$ cells	Cai et al., 2015 (PMID: 25855195, DOI: 10.1523/JNEUROSCI.5083-14.2015); Maass et al., 2016 (PMID: 27918591, DOI: 10.1371/journal.pone.0167286)
Nr2e1	2,4	expressed between P0 and P8	Smeti et al., 2012 (PMID: 22900075, DOI: 10.1371/journal.pone.0042987); Son et al., 2012 (PMID: 22808246, DOI: 10.1371/journal.pone.0040735)
Sp8	2,4	upregulated by FGF signaling during otic placode induction in chick	Yang et al., 2013 (PMID: 23355906, DOI: 10.1371/journal.pone.0055011)
BC048679	2,3	x	
Dcun1d1	2,2	x	
Vgll2	2,2	x	
2310065F04Rik	2,2	x	
*Fgfr3**	2,2	expressed in and regulates the development of pillar and Deiters' cells, gives rise to pillar and Deiters' cells	Hayashi et al., 2007 (PMID: 17117437, DOI: 10.1002/dvdy.21026); Kolla et al., 2020 (PMID: 32404924, DOI: 10.1038/s41467-020-16113-y)
*Crhr1**	2,2	expressed in the outer compartment in adults/expressed in Deiters' cells at P6	Maass et al., 2016 (PMID: 27918591, DOI: 10.1371/journal.pone.0167286); Vetter et al., 2002 (PMID: 12091910, DOI: 10.1038/ng914)
1810062G17Rik	2,0	x	
Mettl11b	2,0	upregulated in cochleae expressing dominant active allele of *Smo*	Chen et al., 2017 (PMID: 29311816, DOI: 10.3389/fnmol.2017.00426)
Gpx6	1,9	involved in age-related hearing loss through oxidative stress	Tanaka et al., 2012 (PMID: 22300951, DOI: 10.1016/j.neurobiolaging.2011.12.027)
C3	1,9	elevated levels in patients with sudden deafness	Nordang et al., 1998 (PMID: 9639471, DOI: 10.1001/archotol.124.6.633)
D230030E09Rik	1,9	x	
Ptpn5	1,9		
*Lgr6**	1,8	*Lgr6-EGFP* is expressed in inner pillar cells from E15.5	Zhang et al., 2015 (PMID: 26029045, DOI: 10.3389/fncel.2015.00165)
Nabp1	1,8	x	
D3Ertd254e	1,7	x	
Mccc1	1,7	x	
*Tectb**	1,7	*Tectb* mutant mice have low frequency hearing loss	Russell et al., 2007 (PMID: 17220887, DOI: 10.1038/nn1828)
Rnf182	1,7	expressed by embryonic and postnatal hair cells	Scheffer et al., 2015 (PMID: 25904789, DOI: 10.1523/JNEUROSCI.5126-14.2015)
Fxyd7	1,7	x	
Slc36a2	1,7	expressed in a supporting cell cluster in P0-P7 cochleae	Scheffer et al., 2015 (PMID: 25904789, DOI: 10.1523/JNEUROSCI.5126-14.2015)
4930512H18Rik	1,7	x	
Pappa2	1,6	differentially expressed along the tonotopic axis of the cochlea at P0-P8	Son et al., 2012 (PMID: 22808246, DOI: 10.1371/journal.pone.0040735)
Jph1	1,6	enriched in spiral ganglion neurons at E12.5	Lu et al., 2011 (PMID: 21795542, DOI: 10.1523/JNEUROSCI.2358-11.2011)
C230034O21Rik	1,6	x	

Gene Symbol	FC	Literature	Reference
Kif6	1,6	expressed by utricular hair cells	Scheffer et al., 2015 (PMID: 25904789, DOI: 10.1523/JNEUROSCI.5126-14.2015)
Acad9	1,6	*ACAD9* patients suffer from mild hearing loss	Collet et al., 2016 (PMID: 26669660, DOI: 10.1038/ejhg.2015.264)
Itgb6	1,6	upregulated in cochleae of *Pten*-cKO mice at E14.5	Kim et al., 2014 (PMID: 24893171, DOI: 10.1371/journal.pone.0097544)
*Ctgf**	1,5	expressed in adult Deiters' cells	Adams 2009 (PMID: 19277783, DOI: 10.1007/s10162-009-0165-z)
Alox15	1,5	expression significantly increased in *Bmi1*$^{-/-}$ *hair cells* after neomycin treatment	Chen et al., 2015 (PMID: 25611380, DOI: 10.1038/cddis.2014.549)
Ctxn3	1,5	belongs to a list of cochlear signature genes detected on macaque or human array chip platform	Mutai et al., 2018 (PMID: 30349143, DOI: 10.1038/s41598-018-33985-9)
Gata6	1,5	x	
Pgm5	1,5	enriched in P1 *Lfng-GFP*$^{+}$ cells, downregulated after DAPT treatment at P0	Maass et al., 2016 (PMID: 27918591, DOI: 10.1371/journal.pone.0167286)

Supplementary Table 3 | List of genes with significantly decreased expression in cochlear ducts of E18.5 *Sox2*$^{CreERT2/+}$*;Tbx2*$^{fl/fl}$ mutants after a single pulse of tamoxifen at E16.5. 4 different pools of control and mutant material were used for the microarray analysis. For genes marked by an asterisk mRNA or protein expression data are available in the literature. E, embryonic day; P, postnatal day.

Gene Symbol	FC	Literature	Reference
Trh	-13,9	enriched in P1 *Lfng-GFP*$^+$ cells compared to P6	Maass et al., 2016 (PMID: 27918591, DOI: 10.1371/journal.pone.0167286)
Prdm12	-11,0	x	
Toporsl	-5,8	x	
*Fgf8**	-3,6	expressed in inner hair cells from E16.5 on	Jacques et al., 2007 (PMID: 17634195, DOI: 10.1242/dev.02874)
Wfdc12	-3,4	x	
*Pkd2l1**	-3,4	expressed in hair cells, enriched in *Lfng-GFP*$^+$ cells at P1	Wu et al., 2016 (PMID: 27196058, DOI: 10.1371/journal.pone.0155577)
Corin	-3,0	strong likelihood of involvement based on their inner ear expression and mapping position within deafness loci	Guipponi et al., 2008 (PMID: 17918732, DOI: 10.1002/humu.20617)
*Cabp2**	-2,9	A mutation in *CABP2* causes autosomal-recessive hearing impairment, restricted to hair cells (much stronger staining in inner than in uter hair cells)	Picher et al., 2017 (PMID: 28183797, DOI: 10.1073/pnas.1617533114); Schrauwen et al., 2012 (PMID: 22981119, DOI: 10.1016/j.ajhg.2012.08.018)
Slc6a14	-2,7	enriched in P1/6 *Lfng-GFP*$^+$ cells, downregulated upon DAPT treatment, expression significantly increase in response to Notch over-activation	Campbell et al., 2016 (PMID: 26786414, DOI: 10.1038/srep19484); Maass et al., 2016 (PMID: 27918591, DOI: 10.1371/journal.pone.0167286)
Wdr95	-2,5	x	
Dnajc5b	-2,5	belongst to the top 10 differentially expressed genes in inner hair cells, expressed by postnatal hair cells	Liu et al., 2014 (PMID: 25122905, DOI: 10.1523/JNEUROSCI.1690-14.2014); Raul et al., 2019 (PMID: 30865901, DOI: 10.1016/j.celrep.2019.02.053); Scheffer et al., 2015 (PMID: 25904789, DOI: 10.1523/JNEUROSCI.5126-14.2015)
1600029I14Rik	-2,4	x	
Uts2b	-2,4	x	
LOC675947	-2,4	x	
*Fgf20**	-2,3	Fgf20-βGal is strongly expressed in inner phalangeal cells and weaker in pillar cells at P0	Huh et al., 2012 (PMID: 22235191, DOI: 10.1371/journal.pbio.1001231)
Gm35208	-2,2	x	
Il33	-2,1	detected in adult organ of Corti samples	Cai et al., 2014 (PMID: 25311735, DOI: 10.1186/s12974-014-0173-8)
Asic4	-2,1	x	
C030013G03Rik	-1,9	downregulated in cochlear cells upon DAPT treatment	Campbell et al., 2016 (PMID: 26786414, DOI: 10.1038/srep19484)
*Otol1**	-1,9	expressed in inner phalangeal and order cells (inner supporting cells) at E18.5	Deans et al., 2010 (PMID: 20856818, DOI: 10.1371/journal.pone.0012765)
Msc	-1,9	differentially expressed in *Pten*- cKO mice at E14.5	Kim et al., 2014 (PMID: 24893171, DOI: 10.1371/journal.pone.0097544)
Nme9	-1,9	downregulated upon *Jag1* deletion	Chrysostomou et al., 2020 (PMID: 33127852, DOI: 10.1523/JNEUROSCI.1192-20.2020)
Cep41	-1,9	expressed in sensory epithelia of zebrafish saccule	Yao et al., 2020 (PMID: 31883312, DOI: 10.1002/ar.24331)
*Slc17a8**	-1,8	expressed in inner hair cells at P21	Seal et al., 2008 (PMID: 18215623, DOI: 10.1016/j.neuron.2007.11.032)
1700028P14Rik	-1,8	expressed by utricular hair cells	Scheffer et al., 2015 (PMID: 25904789, DOI: 10.1523/JNEUROSCI.5126-14.2015)
Ednrb	-1,8	expressed in intermediate cells of the stria vascularis at P1	Renauld et al., 2021 (PMID: 33484097, DOI: 10.1111/pcmr.12961)
Plekhs1	-1,8	x	
*Gabrg3**	-1,8	expressed in hair cells at P5 with much stronger signal in inner hair cells	Girotto et al., 2014 (PMID: 24454846, DOI: 10.1371/journal.pone.0085352)
4930432J09Rik	-1,7	x	
Myot	-1,7	expressed in embryonic and postnatal hair cells	Scheffer et al., 2015 (PMID: 25904789, DOI: 10.1523/JNEUROSCI.5126-14.2015)

Gene Symbol	FC	Literature	Reference
3830417A13Rik	-1,7	x	
Gm5607	-1,7	x	
Hp	-1,7	x	
Dkk1	-1,7	broadly expressed in avian otic epithelium, expression is significantly upregulated in neonatal *Bmi1* $^{-/-}$ mice	Lu et al., 2017 (PMID: 26843109, DOI: 10.1007/s12035-016-9686-8); Sienknecht and Fekete, 2009 (PMID: 19842206, DOI: 10.1002/cne.22169)
Cfap46	-1,7	x	
Ccdc108	-1,7	enriched in utricular hair cells, expressed by postnatal hair cells	Pisciottano et al., 2019 (PMID: 31137036, DOI: 10.1093/molbev/msz077); Scheffer et al., 2015 (PMID: 25904789, DOI: 10.1523/JNEUROSCI.5126-14.2015)
Pkp1	-1,7	identified in the hair cell sample	Hickox et al., 2017 (PMID: 28039372, DOI: 10.1523/JNEUROSCI.2267-16.2016)
Asic2	-1,6	expression in spiral ganglion neurons increases after birth	Peng et al., 2004 (PMID: 15537887, DOI: 10.1523/JNEUROSCI.3196-04.2004)
*Npy**	-1,6	strongly expressed in inner pillar cells at P1	Kolla et al., 2020 (PMID: 32404924, DOI: 10.1038/s41467-020-16113-y)
CJ238989	-1,6	x	
Adamts18	-1,6	x	
Ackr4	-1,6	x	
Hhla1	-1,6	x	
Hydin	-1,6	expressed by postnatal hair cells	Scheffer et al., 2015 (PMID: 25904789, DOI: 10.1523/JNEUROSCI.5126-14.2015)
Cybrd1	-1,6	downregulated in cochlear explants upon DAPT treatment, differentially expressed along the tonotopic axis at P8	Campbell et al., 2016 (PMID: 26786414, DOI: 10.1038/srep19484); Son et al., 2012 (PMID: 22808246, DOI: 10.1371/journal.pone.0040735)
Rgs9bp	-1,5	x	
Gm6146	-1,5	x	
Gm34653	-1,5	x	

Supplementary Table 4 | List of genes with significantly increased expression in cochlear ducts of E18.5 *Sox2*$^{CreERT2/+}$*;Tbx2*$^{fl/fl}$ mutants after a single pulse of tamoxifen at E16.5. 4 different pools of control and mutant material were used for the microarray analysis. For genes marked by an asterisk mRNA or protein expression data are available in the literature. E, embryonic day; P, postnatal day.

Gene Symbol	FC	Literature	Reference
*Esr1**	7,7	expressed in inner and outer hair cells in 3-month-old mice	Simonoska et al., 2009 (PMID: 19293293, DOI: 10.1677/JOE-09-0060)
Pdzk1ip1	3,7	enriched in Deiters' cells 1 and 2 at P1, is downregulated after DAPT treatment in P0 *Lfng-GFP*$^+$ cells	Kolla et al., 2020 (PMID: 32404924, DOI: 10.1038/s41467-020-16113-y); Maass et al., 2016 (PMID: 27918591, DOI: 10.1371/journal.pone.0167286)
Tal1	3,2	epressed in the cochlea (E16.5-P0), expressed in purified cochlear supporting cells (*Lfng-GFP*) at P1	Maass et al., 2016 (PMID: 27918591, DOI: 10.1371/journal.pone.0167286); Perl et al., 2018 (PMID: 29929553, DOI: 10.1186/s40246-018-0161-7)
Dcun1d1	2,8	x	
1810062G17Rik	2,7	x	
2310065F04Rik	2,4	x	
Aqp8	2,3	expression is downregulated in the otic epithelium from E10 until E13	Miyoshi et al., 2017 (PMID: 28004290, DOI: 10.1007/s10162-016-0607-3)
Clca3a1	2,2	x	
Drd5	2,2	expressed in mature outer hair cells (P11–P13) and spiral ganglion neurons	Maison et al., 2012 (PMID: 22219295, DOI: 10.1523/JNEUROSCI.4720-11.2012); Shrestha et al., 2018 (PMID: 30078709, DOI: 10.1016/j.cell.2018.07.007)
Ren1	2,1	x	
Umodl1	2,0	expressed in *Atoh1-GFP*$^+$ cells at P1, enriched in P6 *Lfng-GFP*$^+$ cells	Cai et al., 2015 (PMID: 25855195, DOI: 10.1523/JNEUROSCI.5083-14.2015); Maass et al., 2016 (PMID: 27918591, DOI: 10.1371/journal.pone.0167286)
*Insm2**	2,0	expressed in outer hair cells at P1, enriched in spiral ganglion neurons at E12.5	Lu et al., 2011 (PMID: 21795542, DOI: 10.1523/JNEUROSCI.2358-11.2011); Wiwatpanit et al., 2018 (PMID: 30305733, DOI: 10.1038/s41586-018-0570-8)
D3Ertd254e	2,0	x	
Clca3a2	2,0	x	
Acad9	1,9	*ACAD9* patients suffer from mild hearing loss	Collet et al. 2016 (PMID: 26669660, DOI: 10.1038/ejhg.2015.264)
Mccc1	1,9	x	
Slc6a1	1,8	expressed by supporting cells	Scheffer et al., 2015 (PMID: 25904789, DOI: 10.1523/JNEUROSCI.5126-14.2015)
4930512H18Rik	1,8	x	
Ccdc88b	1,7	x	
Micalcl	1,7	x	
Trim50	1,7	x	
Gm14461	1,7	expressed by utricular hair cells	Scheffer et al., 2015 (PMID: 25904789, DOI: 10.1523/JNEUROSCI.5126-14.2015)
Sox2ot	1,7	x	
Col28a1	1,7	x	
*Crhr1**	1,6	expressed in the outer compartment in adults/expressed in Deiters' cells at P6	Maass et al., 2016 (PMID: 27918591, DOI: 10.1371/journal.pone.0167286); Vetter et al., 2002 (PMID: 12091910, DOI: 10.1038/ng914)
Fam83a	1,6	x	
*Tectb**	1,6	*Tectb* mutant mice have low frequency hearing loss	Rau et al., 1999 (PMID: 10023815); Russell et al., 2007 (PMID: 17220887, DOI: 10.1038/nn1828)
Gm10248	1,5	x	

Gene Symbol	FC	Literature	Reference
D230030E09Rik	1,5	x	
Fam196b	1,5	x	
Tigd4	1,5	x	
Acvr1c	1,5	x	

Supplementary Table 5 | List of antibodies used in this study.

Antibody	Antigen	Host	Dilution	Order number	Lot number	Company/Source
--- Primary antibodies ---						
rat-anti-BCL11B	BCL11B/CTIP2	rat	1:200	#ab18465	GR3272266-2	abcam
rabbit-anti-CALB2	CALB2/Calretinin	rabbit	1:500	#AB5054	2136562	Sigma-Aldrich
rabbit-anti-CALB2	CALB2/Calretinin	rabbit	1:750	#7697	1893-0114	SWANT
rabbit-anti-CDH1	CDH1/E-Cadherin	rabbit	1:500	----	---	obtained from Rolf Kemler
rat-anti-CDH2	CDH2/N-Cadherin	rat	1:200	#MNCD2-c	---	DSHB, MNCD2 was deposited to the DSHB by Takeichi, M. / Matsunami, H.
rabbit-anti-GLAST	EAAT1/GLAST	rabbit	1:200	#ab416	GR3385864-5	abcam
mouse-anti-GFP	GFP	mouse	1:200	#11814460001	27575600	Roche
rabbit-anti-GFP	GFP	rabbit	1:500	#ab290	GR3222604-1	abcam
mouse-anti-IKZF2	IKZF2/Helios	mouse	1:50	#sc-390357	A3015	Santa Cruz Biotechnology
rabbit-anti-KCNQ4	KCNQ4	rabbit	1:100	#HPA018305	---	Sigma-Aldrich
mouse-anti-MYO6	MYO6	mouse	1:200-1000	#sc-393558	I2718	Santa Cruz Biotechnology
rabbit-anti-MYO7A	MYO7A	rabbit	1:500	#25-6790	10119	Proteus Biosciences
rabbit-anti-NGFR	NGFR/P75^{NTR}	rabbit	1:200-500	#AB1554	2101973	Merck Millipore
mouse-anti-parvalbumin	Parvalbumin	mouse	1:250	MAB1572	3770276	Merck Millipore
Phalloidin-iFluor488	F-actin	---	1:1500	#ab176753	GR3411922-4	abcam
rabbit-anti-PROX1	PROX1	rabbit	1:200-500	#ABIN115666	0811R10-1	antikörper-online.de
rabbit-anti-S100A1	S100A1	rabbit	1:500-2000	#C0318-1	310318	Acris Antibodies
guinea pig-anti-SLC17A8	SLC17A8/VGLUT3	guinea pig	1:200-500	#135204	1-8	Synaptic Systems
rabbit-anti-SLC26A5	SLC26A5/Prestin	rabbit	1:200	#sc-30163	----	Santa Cruz Biotechnology
rabbit-anti-SOX2	SOX2	rabbit	1:200	#ab97959	----	abcam
mouse-anti-TBX2	TBX2	mouse	1:200	#sc-514291 X	C2417	Santa Cruz Biotechnology
rabbit-anti-TBX2	TBX2	rabbit	1:200	#07-318	3030644	Merck Millipore
--- Secondary antibodies ---						
donkey anti-mouse IgG (Alexa405)	AlexaFluor-405	donkey	1:200	#ab175658	GR3258168-3	abcam
donkey-anti-mouse IgG (Alexa488)	AlexaFluor-488	donkey	1:200	#A-21202	1975519	Invitrogen
donkey anti-sheep IgG (Alexa488)	AlexaFluor-488	donkey	1:200	#ab150177	GR3232735-4	abcam
goat-anti-mouse IgG (Alexa555)	AlexaFluor-555	goat	1:500	#A-21422	2090527	Invitrogen
goat-anti-rabbit IgG (Alexa488)	AlexaFluor-488	goat	1:200	#A-11034	2018207	Invitrogen
donkey anti-goat IgG (Alexa555)	AlexaFluor-555	donkey	1:200	#ab150134	GR3239464-1	abcam
goat-anti-rabbit IgG (Alexa555)	AlexaFluor-555	goat	1:200	#A-21428	2011559	Invitrogen
donkey-anti-rabbit IgG (Alexa647)	AlexaFluor-647	donkey	1:200	#ab150075	GR289683-1	abcam
sheep-anti-mouse IgG (Alexa647)	AlexaFluor-647	sheep	1:200	#515-605-003	130743	Dianova
Biotin-conjug. donkey-anti-rat FAB	Biotin	donkey	1:200	#712-067-003	137943	Dianova
Biotin-conjug. goat-anti-mouse IgG	Biotin	goat	1:200	#115-065-003	130488	Jackson Immuno Research
Biotin-conjug. goat-anti-rabbit FAB	Biotin	goat	1:200	#111-067-003	131076	Jackson Immuno Research
Biotin-conjug. goat-anti-rat	Biotin	goat	1:200	#112-065-003	80972	Jackson Immuno Research
goat-anti-guinea pig (Cy3)	Cy3	goat	1:200	#106-165-003	67623	Dianova
donkey-anti-mouse FAB	none	donkey	1:100	#715-007-003	109583	Dianova
goat-anti-rabbit FAB	none	goat	1:50	#111-007-003	135805	Dianova
goat-anti-guinea pig IgG	none	goat	1:200	#106-005-003	139376	Dianova
sheep-anti-rabbit IgG	none	sheep	1:200	#ShxRB-003-D	66-6-061318	Dianova
Streptavidin-HRP	HRP	none	1:200	#434323	TC265875	Invitrogen
Streptavidin-AlexaFluor647	AlexaFluor-647	none	1:200	#016-600-084	135095	Dianova
Streptavidin-Dylight549	Dylight-549	none	1:200	#016-500-084	95559	Dianova

Supplementary Table 6 | List of primers used for synthesis of DNA-templates for in vitro transcription of antisense RNA probes. A T7-promoter sequence (TAATACGACTCACTATAGGG) was added to the 5' end of the reverse primer.

Gene	Forward primer	Reverse Primer	Length of the template	Source
Ccdc88b	AGCTCAGATGCTACTGGCCG	ACGCCTAAGCCGTCTGTCC	958 bp	cDNA
Crhr1	GCATCAAGCAGTCCACAGCAG	CGGCTCCTCAGCTCTCCTAC	971 bp	genomic DNA
Ctgf	GCACAGAACCACCACTCTGC	AGCTTCGTGTCCTACCTATGGTG	902 bp	genomic DNA
Insm1	AGTGCCACCTGTGCCCAGTG	AGTCTTCCGGGATATACTGAGGCAG	900 bp	genomic DNA
Insm2	CGAGTGCGACAAGGTCTTCA	TAGACAGACTTGCAGGCGT	1121 bp	genomic DNA
Itgb6	TAAGAACGTGACCTACAAGC	TGAAGACTGTAGTTGAGTGC	1030 bp	genomic DNA
Jph1	ATGATTGTCCTGGTCATGCTGTTG	CCAGTGTGTTCTCATCCAGCC	930 bp	genomic DNA
Lgr6	GCATCCGCCTTGCTGTGTGG	TTGGGTGGAGTCGCAGGAGC	917 bp	genomic DNA
Mettl11b	TGAAGGACAATGTGGCACGG	ATTCAAGGAATGAGGACCAGATGC	1010 bp	genomic DNA
Nabp1	TAGCCGCACCTGCCTCAGTATG	ATGGCAGGCAGACACTCTAGGG	1113 bp	cDNA
Nr2e1	AGCTTTACGGTCAATTAGCCCA	TCCTTGGACGGAGGTTCTCA	958 bp	genomic DNA
Pdzk1ip1	AACACCAGCAGTGGACCG	AGGAGCTGAGCCACCCTT	628 bp	cDNA
Ptpn5	ATGCGAACAGTACCAGTTTGTG	TCCACATCCTACTCTTGGAGG	827 bp	genomic DNA
Rnf182	TCGACTCTGTCACCACCGTG	AGAGGCAGCGGACTTCAGAG	856 bp	genomic DNA
Slc6a1	CTGGCACTCTGGACTGGCTG	TGTCTCCGCTGGCTTCACC	995 bp	genomic DNA
Slc36a2	GTGACAGAGCCTCTCCTCGC	CAAGTTACAAGGTCAAGACTCAGG	825 bp	genomic DNA
Tal1	TGTGGCAGCTCTCTGGATGG	GGCTGCTGACTTGGTCCTCAC	826 bp	genomic DNA
Tectb	TGTAGCAGCTCTCTCCAAGGC	CCAGTTCTGAGAGTGTGC	1022 bp	genomic DNA
Otol1	TGGCTTCATTGGAGAGCCTG	TGAACCTCTGGGTGCCATTC	1108 bp	genomic DNA

Supplementary References

Adams, J. C. Immunocytochemical traits of type IV fibrocytes and their possible relations to cochlear function and pathology. *JARO - J. Assoc. Res. Otolaryngol.* **10**, 369–382 (2009).

Basch, M. L. *et al.* Fine-tuning of Notch signaling sets the boundary of the organ of Corti and establishes sensory cell fates. *Elife* **5**, 1–23 (2016).

Brand, Y. *et al.* Neural cell adhesion molecule NrCAM is expressed in the mammalian inner ear and modulates spiral ganglion neurite outgrowth in an in vitro alternate choice assay. *J. Mol. Neurosci.* **55**, 836–44 (2015).

Brown, S. T., Wang, J. & Groves, A. K. Dlx gene expression during chick inner ear development. *J. Comp. Neurol.* **483**, 48–65 (2005).

Cai, Q. *et al.* Molecular profile of cochlear immunity in the resident cells of the organ of Corti. *J. Neuroinflammation* **11**, 173 (2014).

Cai, T. *et al.* Characterization of the Transcriptome of Nascent Hair Cells and Identification of Direct Targets of the Atoh1 Transcription Factor. *J. Neurosci.* **35**, 5870–5883 (2015).

Campbell, D. P., Chrysostomou, E. & Doetzlhofer, A. Canonical Notch signaling plays an instructive role in auditory supporting cell development. *Sci. Rep.* **6**, 19484 (2016).

Chen, Y. *et al.* Bmi1 regulates auditory hair cell survival by maintaining redox balance. *Cell Death Dis.* **6**, e1605 (2015).

Chen, Y. *et al.* Hedgehog Signaling Promotes the Proliferation and Subsequent Hair Cell Formation of Progenitor Cells in the Neonatal Mouse Cochlea. *Front. Mol. Neurosci.* **10**, 426 (2017).

Chrysostomou, E. *et al.* The Notch Ligand Jagged1 Is Required for the Formation, Maintenance, and Survival of Hensen's Cells in the Mouse Cochlea. *J. Neurosci.* **40**, 9401–9413 (2020).

Collet, M. *et al.* High incidence and variable clinical outcome of cardiac hypertrophy due to ACAD9 mutations in childhood. *Eur. J. Hum. Genet.* **24**, 1112–6 (2016).

Deans, M. R., Peterson, J. M. & Wong, G. W. Mammalian otolin: A multimeric glycoprotein specific to the inner ear that interacts with otoconial matrix protein otoconin-90 and cerebellin-1. *PLoS One* **5**, 1–15 (2010).

Girotto, G. *et al.* Expression and replication studies to identify new candidate genes involved in normal hearing function. *PLoS One* **9**, e85352 (2014).

Guipponi, M. *et al.* An integrated genetic and functional analysis of the role of type II transmembrane serine proteases (TMPRSSs) in hearing loss. *Hum. Mutat.* **29**, 130–41 (2008).

Harley, R. J. *et al.* Neuronal cell adhesion molecule (NrCAM) is expressed by sensory cells in the cochlea and is necessary for proper cochlear innervation and sensory domain patterning during development. *Dev. Dyn.* **247**, 934–950 (2018).

Hayashi, T., Cunningham, D. & Bermingham-McDonogh, O. Loss of Fgfr3 leads to excess hair cell development in the mouse organ of Corti. *Dev. Dyn.* **236**, 525–533 (2007).

Hickox, A. E. *et al.* Global Analysis of Protein Expression of Inner Ear Hair Cells. *J. Neurosci.* **37**, 1320–1339 (2017).
Huebner, A. K. *et al.* Nonsense Mutations in SMPX, Encoding a Protein Responsive to Physical Force, Result in X-Chromosomal Hearing Loss. *Am. J. Hum. Genet.* **88**, 621–627 (2011).

Huh, S.-H., Jones, J., Warchol, M. E. & Ornitz, D. M. Differentiation of the lateral compartment of the cochlea requires a temporally restricted FGF20 signal. *PLoS Biol.* **10**, 1–12 (2012).

Jacques, B. E., Montcouquiol, M. E., Layman, E. M., Lewandoski, M. & Kelley, M. W. Fgf8 induces pillar cell fate and regulates cellular patterning in the mammalian cochlea. *Development* **134**, 3021–3029 (2007).

Kaiser, M. *et al.* Regulation of otocyst patterning by Tbx2 and Tbx3 is required for inner ear morphogenesis in the mouse. *Development* **148**, (2021).

Kanaya, E. *et al.* Expression of the Olig gene family in the developing mouse inner ear. *Gene Expr. Patterns* **17**, 79–86 (2015).

Katayama, K. *et al.* Disorganized innervation and neuronal loss in the inner ear of Slitrk6-deficient mice. *PLoS One* **4**, e7786 (2009).

Kim, H. J. *et al.* Patterns of gene expression associated with Pten deficiency in the developing inner ear. *PLoS One* **9**, e97544 (2014).

Kim, Y. J. *et al.* EphA7 regulates spiral ganglion innervation of cochlear hair cells. *Dev. Neurobiol.* **76**, 452–69 (2016).

Kolla, L. *et al.* Characterization of the development of the mouse cochlear epithelium at the single cell level. *Nat. Commun.* **11**, 2389 (2020).

Lewis, M. A. *et al.* Exploring regulatory networks of miR-96 in the developing inner ear. *Sci. Rep.* **6**, 23363 (2016).
Li, C. *et al.* Comprehensive transcriptome analysis of cochlear spiral ganglion neurons at multiple ages. *Elife* **9**, (2020).

Lingle, C. J. *et al.* LRRC52 regulates BK channel function and localization in mouse cochlear inner hair cells. *Proc. Natl. Acad. Sci.* **116**, 18397–18403 (2019).

Liu, H. *et al.* Characterization of Transcriptomes of Cochlear Inner and Outer Hair Cells. *J. Neurosci.* **34**, 11085–11095 (2014).

Lu, C. C., Appler, J. M., Houseman, E. A. & Goodrich, L. V. Developmental profiling of spiral ganglion neurons reveals insights into auditory circuit assembly. *J. Neurosci.* **31**, 10903–18 (2011).

Lu, X. *et al.* Bmi1 Regulates the Proliferation of Cochlear Supporting Cells Via the Canonical Wnt Signaling Pathway. *Mol. Neurobiol.* **54**, 1326–1339 (2017).

Maass, J. C. *et al.* Transcriptomic analysis of mouse cochlear supporting cell maturation reveals large-scale changes in Notch responsiveness prior to the onset of hearing. *PLoS One* **11**, 167286 (2016).

Maison, S. F. *et al.* Dopaminergic signaling in the cochlea: receptor expression patterns and deletion phenotypes. *J. Neurosci.* **32**, 344–355 (2012).

Mendus, D. *et al.* Thrombospondins 1 and 2 are important for afferent synapse formation and function in the inner ear. *Eur. J. Neurosci.* **39**, 1256–67 (2014).

Miyoshi, T. *et al.* Quantitative Analysis of Aquaporin Expression Levels during the Development and Maturation of the Inner Ear. *J. Assoc. Res. Otolaryngol.* **18**, 247–261 (2017).

Mutai, H. *et al.* Gene expression dataset for whole cochlea of Macaca fascicularis. *Sci. Rep.* **8**, 15554 (2018).

Nordang, L., Laurent, C. & Mollnes, T. E. Complement activation in sudden deafness. *Arch. Otolaryngol. Head. Neck Surg.* **124**, 633–6 (1998).

Odeh, H. *et al.* Mutations in Grxcr1 Are The Basis for Inner Ear Dysfunction in the Pirouette Mouse. *Am. J. Hum. Genet.* **86**, 148–160 (2010).

Peng, B.-G. *et al.* Acid-sensing ion channel 2 contributes a major component to acid-evoked excitatory responses in spiral ganglion neurons and plays a role in noise susceptibility of mice. *J. Neurosci.* **24**, 10167–75 (2004).

Perl, K., Shamir, R. & Avraham, K. B. Computational analysis of mRNA expression profiling in the inner ear reveals candidate transcription factors associated with proliferation, differentiation, and deafness. *Hum. Genomics* **12**, 30 (2018).

Picher, M. M. *et al.* Ca2+-binding protein 2 inhibits Ca2+-channel inactivation in mouse inner hair cells. *Proc. Natl. Acad. Sci. U. S. A.* **114**, E1717–E1726 (2017).

Pisciottano, F. *et al.* Inner Ear Genes Underwent Positive Selection and Adaptation in the Mammalian Lineage. *Mol. Biol. Evol.* **36**, 1653–1670 (2019).

Ranum, P. T. *et al.* Insights into the Biology of Hearing and Deafness Revealed by Single-Cell RNA Sequencing. *Cell Rep.* **26**, 3160-3171.e3 (2019).

Rau, A., Legan, P. K. & Richardson, G. P. Tectorin mRNA expression is spatially and temporally restricted during mouse inner ear development. *J. Comp. Neurol.* **405**, 271–280 (1999).

Reisinger, E., Meintrup, D., Oliver, D. & Fakler, B. Gene expression associated with the onset of hearing detected by differential display in rat organ of Corti. *Eur. J. Hum. Genet.* **18**, 1327–32 (2010).

Renauld, J. M., Davis, W., Cai, T., Cabrera, C. & Basch, M. L. Transcriptomic analysis and ednrb expression in cochlear intermediate cells reveal developmental differences between inner ear and skin melanocytes. *Pigment Cell Melanoma Res.* **34**, 585–597 (2021).

Russell, I. J. *et al.* Sharpened cochlear tuning in a mouse with a genetically modified tectorial membrane. *Nat. Neurosci.* **10**, 215–23 (2007).

Scheffer, D. I., Shen, J., Corey, D. P. & Chen, Z.-Y. Gene Expression by Mouse Inner Ear Hair Cells during Development. *J. Neurosci.* **35**, 6366–6380 (2015).

Schrauwen, I. *et al.* A mutation in CABP2, expressed in cochlear hair cells, causes autosomal-recessive hearing impairment. *Am. J. Hum. Genet.* **91**, 636–45 (2012).

Seal, R. P. *et al.* Sensorineural Deafness and Seizures in Mice Lacking Vesicular Glutamate Transporter 3. *Neuron* **57**, 263–275 (2008).

Sherrill, H. E. *et al.* Pou4f1 Defines a Subgroup of Type I Spiral Ganglion Neurons and Is Necessary for Normal Inner Hair Cell Presynaptic Ca2+ Signaling. *J. Neurosci.* **39**, 5284–5298 (2019).

Shrestha, B. R. *et al.* Sensory Neuron Diversity in the Inner Ear Is Shaped by Activity. *Cell* **174**, 1229-1246.e17 (2018).

Sienknecht, U. J. & Fekete, D. M. Mapping of Wnt, frizzled, and Wnt inhibitor gene expression domains in the avian otic primordium. *J. Comp. Neurol.* **517**, 751–64 (2009).

Simmons, D. D., Tong, B., Schrader, A. D. & Hornak, A. J. Oncomodulin identifies different hair cell types in the mammalian inner ear. *J. Comp. Neurol.* **518**, 3785–3802 (2010).

Simonoska, R. *et al.* Inner ear pathology and loss of hearing in estrogen receptor-beta deficient mice. *J. Endocrinol.* **201**, 397–406 (2009).

Smeti, I., Assou, S., Savary, E., Masmoudi, S. & Zine, A. Transcriptomic analysis of the developing and adult mouse cochlear sensory epithelia. *PLoS One* **7**, e42987 (2012).

Son, E. J. *et al.* Developmental Gene Expression Profiling along the Tonotopic Axis of the Mouse Cochlea. *PLoS One* **7**, e40735 (2012).

Tanaka, C. *et al.* Expression pattern of oxidative stress and antioxidant defense-related genes in the aging Fischer 344/NHsd rat cochlea. *Neurobiol. Aging* **33**, 1842.e1–14 (2012).

Uchida, Y., Sugiura, S., Nakashima, T., Ando, F. & Shimokata, H. Endothelin-1 gene polymorphism and hearing impairment in elderly Japanese. *Laryngoscope* **119**, 938–943 (2009).

Vetter, D. E. *et al.* Urocortin-deficient mice show hearing impairment and increased anxiety-like behavior. **31**, 363 (2002).

von Bartheld, C. S. *et al.* Expression of nerve growth factor (NGF) receptors in the developing inner ear of chick and rat. *Development* **113**, 455–70 (1991).

Wiwatpanit, T. *et al.* Trans-differentiation of outer hair cells into inner hair cells in the absence of INSM1. *Nature* **563**, 691–695 (2018).

Wu, X. *et al.* Hair-cell mechanotransduction persists in TRP channel knockout mice. *PLoS One* **11**, e0155577 (2016).

Yang, L. *et al.* Analysis of FGF-Dependent and FGF-Independent Pathways in Otic Placode Induction. *PLoS One* **8**, (2013).

Yao, Q. *et al.* Transcriptomic Analyses of Inner Ear Sensory Epithelia in Zebrafish. *Anat. Rec. (Hoboken).* **303**, 527–543 (2020).

Yoon, H., Lee, D. J., Kim, M. H. & Bok, J. Identification of genes concordantly expressed with Atoh1 during inner ear development. *Anat. Cell Biol.* **44**, 69–78 (2011).

Zhang, Y. *et al.* Dynamic expression of Lgr6 in the developing and mature mouse cochlea. *Frontiers in Cellular Neuroscience* **9**, 165 (2015).

Zwaenepoel, I. *et al.* Otoancorin, an inner ear protein restricted to the interface between the apical surface of sensory epithelia and their overlying acellular gels, is defective in autosomal recessive deafness DFNB22. *Proc. Natl. Acad. Sci.* **99**, 6240–6245 (2002).
