# Supplementary material for: TBX2 specifies and maintains inner hair and supporting cell fate in the Organ of Corti
Source: Nat Commun. 2022 Dec 9;13:7628. doi: 10.1038/s41467-022-35214-4 (PMC9734556; doi:10.1038/s41467-022-35214-4)
Supplement: Supplementary file 3 — Reporting Summary [file 41467_2022_35214_MOESM3_ESM.pdf]

nature portfolio

Corresponding author(s): Andreas Kispert, Mark-Oliver Trowe

Last updated by author(s): Nov 16, 2022

Reporting Summary

Nature Portfolio wishes to improve the reproducibility of the work that we publish. This form provides structure for consistency and transparency in reporting. For further information on Nature Portfolio policies, see our Editorial Policies and the Editorial Policy Checklist.

Statistics

For all statistical analyses, confirm that the following items are present in the figure legend, table legend, main text, or Methods section.

n/a	Confirmed	
☐	☒	The exact sample size (*n*) for each experimental group/condition, given as a discrete number and unit of measurement
☐	☒	A statement on whether measurements were taken from distinct samples or whether the same sample was measured repeatedly
☐	☒	The statistical test(s) used AND whether they are one- or two-sided<br>*Only common tests should be described solely by name; describe more complex techniques in the Methods section.*
☒	☐	A description of all covariates tested
☐	☒	A description of any assumptions or corrections, such as tests of normality and adjustment for multiple comparisons
☐	☒	A full description of the statistical parameters including central tendency (e.g. means) or other basic estimates (e.g. regression coefficient) AND variation (e.g. standard deviation) or associated estimates of uncertainty (e.g. confidence intervals)
☐	☒	For null hypothesis testing, the test statistic (e.g. *F*, *t*, *r*) with confidence intervals, effect sizes, degrees of freedom and *P* value noted<br>*Give P values as exact values whenever suitable.*
☒	☐	For Bayesian analysis, information on the choice of priors and Markov chain Monte Carlo settings
☒	☐	For hierarchical and complex designs, identification of the appropriate level for tests and full reporting of outcomes
☐	☒	Estimates of effect sizes (e.g. Cohen's *d*, Pearson's *r*), indicating how they were calculated

Our web collection on statistics for biologists contains articles on many of the points above.

Software and code

Policy information about availability of computer code

Data collection	Images of sections were acquired using a Leica DM5000 microscope with a Leica DFC300FX digital camera and Leica Firecam for Mac 1.9 software, or a Leica DM6000 microscope with a Leica DFC350FX digital camera and Leica Application Suite X (3.0.1) software.<br>Images of cochlea whole-mount specimen were acquired using the confocal laser scanning microscope Leica TCS SP8 with Leica Application Suite X 3.5.5 software.<br>Microarray data were obtained using the Agilent Micro Array Scanner G2565CA system.
Data analysis	MacVector software (version 16.0.8)<br>FIJI/ImageJ 2.3.0 (NIH)<br>Microsoft Excel 16.16.27 (Microsoft)<br>Photoshop CS4 (Adobe)<br>Leica Application Suite X 3.0.1 (Leica)<br>Prism7 (Graphpad)<br>JASP (0.13.1) (jasp-stats.org)<br>Significance Analysis of Microarrays 5.0 (SAM, Stanford University, USA)

For manuscripts utilizing custom algorithms or software that are central to the research but not yet described in published literature, software must be made available to editors and reviewers. We strongly encourage code deposition in a community repository (e.g. GitHub). See the Nature Portfolio guidelines for submitting code & software for further information.

Data

Policy information about availability of data

All manuscripts must include a data availability statement. This statement should provide the following information, where applicable:

- Accession codes, unique identifiers, or web links for publicly available datasets
- A description of any restrictions on data availability
- For clinical datasets or third party data, please ensure that the statement adheres to our policy

Source data for this study are provided with this paper. All relevant data in this study are available within the article, Supplementary Data, or Source Data. Microarray data have been deposited in Gene Expression Omnibus under the accession numbers GSE180500 (https://www.ncbi.nlm.nih.gov/geo/query/acc.cgi?acc=GSE180500) and GSE180501 (https://www.ncbi.nlm.nih.gov/geo/query/acc.cgi?acc=GSE180501).

Field-specific reporting

Please select the one below that is the best fit for your research. If you are not sure, read the appropriate sections before making your selection.

- [x] Life sciences
- [ ] Behavioural & social sciences
- [ ] Ecological, evolutionary & environmental sciences

For a reference copy of the document with all sections, see nature.com/documents/nr-reporting-summary-flat.pdf

Life sciences study design

All studies must disclose on these points even when the disclosure is negative.

Sample size	No statistical methods were used to predetermine sample size. We used a minimum of 3 animals for each analyses based on the availability of our KO and ME animals. For statistical analyses, we calculated all analyses based on n = the number of cochleae used.<br>For E14.5 microarray experiments we used 4 mutant pools and 4 control pools with 23-27 cochlea each.<br>For E18.5 microarray experiments we used 4 mutant pools and 4 control pools with 16-20 organs of Corti each.
Data exclusions	No data were excluded from the statistical analyses in this study.
Replication	All attempts at replication were successful. The sample sizes are indicated in the figure legends and methods.
Randomization	Experimental design was not random. All animals were assigned to groups based on their genotype.
Blinding	The experiments were not blinded due to the obvious phenotype.

Reporting for specific materials, systems and methods

We require information from authors about some types of materials, experimental systems and methods used in many studies. Here, indicate whether each material, system or method listed is relevant to your study. If you are not sure if a list item applies to your research, read the appropriate section before selecting a response.

Materials & experimental systems

n/a	Involved in the study	
	x	Antibodies
x		Eukaryotic cell lines
x		Palaeontology and archaeology
	x	Animals and other organisms
x		Human research participants
x		Clinical data
x		Dual use research of concern

Methods

n/a	Involved in the study	
x		ChIP-seq
x		Flow cytometry
x		MRI-based neuroimaging

Antibodies

Antibodies used	primary antibodies:<br>rat-anti-BCL11B (used at 1:200, catalog #ab18465, Lot #GR3272266-2, abcam),<br>rabbit-anti-CALB2 (used at 1:500, catalog #AB5054, Lot #2136562, Sigma-Aldrich),<br>rabbit-anti-CALB2 (used at 1:750, catalog #7697, LOT #1893-0114, SWANT),<br>rabbit-anti-CHD1 (used at 1:500, a gift from Rolf Kemler),<br>rat-anti-CHD2 (used at 1:200, catalog #MNCD2-c, Developmental Studies Hybridoma Bank),<br>mouse-anti-GFP (used at 1:200, catalog #11814460001, Lot #27575600, Roche),

rabbit-anti-GFP (used at 1:500, catalog #ab290, Lot #GR3222604-1, abcam),
rabbit-anti-GLAST/EAAT1 (used at 1:200, catalog #ab416, LOT #GR3385864-5, abcam),
mouse-anti-IKZF2 (used at 1:50, catalog #sc-390357, Lot #A3015, Santa Cruz Biotechnology),
rabbit-anti-KCNQ4 (used at 1:100, catalog #HPA018305, Sigma-Aldrich),
mouse-anti-MYO6 (used at 1:200-1000, catalog #sc-393558, Lot #I2718 , Santa Cruz Biotechnology),
rabbit-anti-MYO7A (used at 1:500, catalog #25-6790, Lot #10119, Proteus Biosciences),
rabbit-anti-NGFR (used at 1:200-500, catalog #AB1554, Lot #2101973, Merck Millipore),
mouse-anti-PARVALBUMIN (used at 1:250, catalog #MAB1572, LOT #3770276, Merck Millipore),
Phalloidin-iFluor488 (used at 1:1500, catalog #ab176753, LOT #GR3411922-4, abcam),
rabbit-anti-PROX1 (used at 1:200-500, catalog #ABIN115666, Lot #0811R10-1, antikörper-online.de),
rabbit-anti-S100A1 (used at 1:500-2000, catalog #C0318-1, Lot #310318, Acris Antibodies),
guinea pig-anti-SLC17A8 (used at 1:200-500, catalog #135204, Lot #1-8, Synaptic Systems),
rabbit-anti-SLC26A5 (used at 1:200, catalog #sc-30163, Santa Cruz Biotechnology),
rabbit-anti-SOX2 (used at 1:200, catalog #ab97959, abcam),
mouse-anti-TBX2 (used at 1:200, catalog #sc-514291 X, Lot #C2417, Santa Cruz Biotechnology),
rabbit-anti-TBX2 (used at 1:200, catalog #07-318, Lot #3030644, Merck Millipore),
sheep-anti-DIG, AP-conjugated (used at 1:4000, catalog # 11093274910, Lot #32871920, Roche)

secondary antibodies:
Antibody (used at Dilution, catalog Order number, Lot #Lot number, Company),
donkey anti-mouse IgG (Alexa405) (used at 1:200, catalog #ab175658, Lot #GR3258168-3, abcam),
donkey-anti-mouse IgG (Alexa488) (used at 1:200, catalog #A-21202, Lot #1975519, Invitrogen),
donkey anti-sheep IgG (Alexa488) (used at 1:200, catalog #ab150177, Lot #GR3232735-4, abcam),
goat-anti-mouse IgG (Alexa555) (used at 1:500, catalog #A-21422, Lot #2090527, Invitrogen),
goat-anti-rabbit IgG (Alexa488) (used at 1:200, catalog #A-11034, Lot #2018207, Invitrogen),
donkey anti-goat IgG (Alexa555) (used at 1:200, catalog #ab150134, Lot #GR3239464-1, abcam),
goat-anti-rabbit IgG (Alexa555) (used at 1:200, catalog #A-21428, Lot #2011559, Invitrogen),
donkey-anti-rabbit IgG (Alexa647) (used at 1:200, catalog #ab150075, Lot #GR289683-1, abcam),
sheep-anti-mouse IgG (Alexa647) (used at 1:200, catalog #515-605-003, Lot #130743, Dianova),
Biotin-conjug. donkey-anti-rat FAB (used at 1:200, catalog #712-067-003, Lot #137943, Dianova),
Biotin-conjug. goat-anti-mouse IgG (used at 1:200, catalog #115-065-003, Lot #130488, Jackson Immuno Research),
Biotin-conjug. goat-anti-rabbit FAB (used at 1:200, catalog #111-067-003, Lot #131076, Jackson Immuno Research),
Biotin-conjug. goat-anti-rat (used at 1:200, catalog #112-065-003, Lot #80972, Jackson Immuno Research),
goat-anti-guinea pig (Cy3) (used at 1:200, catalog #106-165-003, Lot #67623, Dianova),
donkey-anti-mouse FAB (used at 1:100, catalog #715-007-003, Lot #109583, Dianova),
goat-anti-rabbit FAB (used at 1:50, catalog #111-007-003, Lot #135805, Dianova),
goat-anti-guinea pig IgG (used at 1:200, catalog #106-005-003, Lot #139376, Dianova),
sheep-anti-rabbit IgG (used at 1:200, catalog #ShxRB-003-D, Lot #66-6-061318, Dianova),
Streptavidin-HRP (used at 1:200, catalog #434323, Lot #TC265875, Invitrogen),
Streptavidin-AlexaFluor647 (used at 1:200, catalog #016-600-084, Lot #135095, Dianova),
Streptavidin-Dylight549 (used at 1:200, catalog #016-500-084, Lot #95559, Dianova),

Validation

rabbit-anti-CHD1 (a gift from Rolf Kemler) was validated in (Vestweber, D., Kemler, R., 1984. Rabbit antiserum against a purified surface glycopro- tein decompacts mouse preimplantation embryos and reacts with specific adult tissues. Exp. Cell Res. 152, 169–178.)
rat-anti-CHD2 (#MNCD2-c, DSHB) was validated in "Fetal brain subdivisions defined by R- and E-cadherin expressions: evidence for the role of cadherin activity in region-specific, cell-cell adhesion., Takeichi M, Developmental biology 172.2 (1995 Dec): 466-78.)

All commercially available antibodies were validated by the manufacturer.
rat-anti-BCL11B (#ab18465, abcam), [https://www.abcam.com/ctip2-antibody-25b6-ab18465.html]
rabbit-anti-CALB2 (#AB5054, Sigma-Aldrich), [https://www.sigmaaldrich.com/DE/de/product/mm/ab5054]
rabbit-anti-CALB2 (#7697, SWANT), [https://www.swant.com/pdfs/Rabbit_anti_calretinin_7697.pdf]
mouse-anti-GFP (#11814460001, Roche), [https://www.sigmaaldrich.com/DE/de/product/roche/11814460001]
rabbit-anti-GFP (#ab290, abcam), [https://www.abcam.com/gfp-antibody-ab290.html]
rabbit-anti-GLAST/EAAT1 (#ab416, abcam), [https://www.abcam.com/eaat1-antibody-ab416.html]
mouse-anti-IKZF2 (#sc-390357, Santa Cruz Biotechnology), [https://www.scbt.com/de/p/helios-antibody-e-7]
rabbit-anti-KCNQ4 (#HPA018305, Sigma-Aldrich), [https://www.sigmaaldrich.com/DE/de/product/sigma/hpa018305]
mouse-anti-MYO6 (#sc-393558, Santa Cruz Biotechnology), [https://www.scbt.com/de/p/myosin-vi-antibody-a-9]
rabbit-anti-MYO7A (#25-6790, Proteus Biosciences), [http://www.proteus-biosciences.com/product/view/myosin-viia-248.aspx]
rabbit-anti-NGFR (#AB1554, Merck Millipore), [https://www.merckmillipore.com/DE/de/product/Anti-Nerve-Growth-Factor-Receptor-Antibody-p75]
mouse-anti-PARVALBUMIN (MAB1572, Merck Millipore), [https://www.merckmillipore.com/DE/de/product/Anti-Parvalbumin-Antibody,MM_NF-MAB1572]
Phalloidin-iFluor488 (#ab176753, abcam), [https://www.abcam.com/phalloidin-ifluor-488-reagent-ab176753.html]
rabbit-anti-PROX1 (#ABIN115666, antikörper-online.de), [https://www.antikoerper-online.de/antibody/3032348/anti-Prospero+Homeobox+1+PROX1+AA+492-522+antibody/]
rabbit-anti-S100A1 (#C0318-1, Acris Antibodies), [https://docs.aatbio.com/products/pis/C0318.pdf]
guinea pig-anti-SLC17A8 (#135204, Synaptic Systems), [https://sysy.com/product/135204]
rabbit-anti-SLC26A5 (#sc-30163, Santa Cruz Biotechnology), [https://datasheets.scbt.com/sc-30163.pdf]

rabbit-anti-SOX2 (#ab97959, abcam), [https://www.abcam.com/sox2-antibody-ab97959.html]
mouse-anti-TBX2 (#sc-514291 X, Santa Cruz Biotechnology), [https://www.scbt.com/de/p/tbx2-antibody-d-3]
rabbit-anti-TBX2 (#07-318, Merck Millipore), [https://www.merckmillipore.com/DE/de/product/Anti-TBX2-Antibody,MM_NF-07-318]

Animals and other organisms

Policy information about studies involving animals; ARRIVE guidelines recommended for reporting animal research

Laboratory animals	All information for laboratory animals used are included in the methods section. Up to four mice per cage were housed with ad libitum access to food and water under conditions of regulated temperature (22°C) and humidity (50%) and a 12-h light/ dark cycle at the central animal laboratory of Hannover Medical School.<br>All mouse alleles employed in this study have previously been described and were maintained on an NMRI genetic background. A conditional floxed allele of Tbx2 [Tbx2tm2.1Vmc, synonym: Tbx2fl] was provided by Vincent Christoffels; an allele with insertion of the human TBX2 gene at the Hprt locus [Hprttm2(CAG-TBX2,-EGFP)Akis, synonym: HprtTBX2] was generated in house. The double fluorescent Cre reporter line Gt(ROSA)26Sortm4(ACTB-tdTomato,-EGFP)Luo/J [#007576, synonym: R26mTmG], the tamoxifen-inducible CreERT2 driver lines Sox2CreERT2 [#017593, Sox2tm1(cre/ERT2)Hoch] and Atoh1-CreERT2 [#007684, Tg(Atoh1-cre/Esr1*)14Fsh] were obtained from The Jackson Laboratory.<br>Embryos for Tbx2/TBX2 expression analyses were derived from matings of NMRI wild-type mice.<br>For generation of loss-of-function mutants Sox2CreERT2/+;Tbx2fl/fl males were mated with Tbx2fl/fl;R26mTmG/mTmG females to obtain Sox2CreERT2/+;Tbx2fl/fl;R26mTmG/+ (Sox2-Tbx2LOF) mice, and Atoh1-CreERT2/+;Tbx2fl/fl males with Tbx2fl/fl;R26mTmG/mTmG females to obtain Atoh1-CreERT2/+;Tbx2fl/fl;R26mTmG/+ (Atoh1-Tbx2LOF) mice. For generation of misexpression mutants Sox2CreERT2/+ males were mated with HprtTBX2/TBX2 females to obtain Sox2CreERT2/+;HprtTBX2/Y(+) (Sox2-TBX2ME) mice, and Atoh1-CreERT2/+ males with HprtTBX2/TBX2 females to obtain Atoh1-CreERT2/+; HprtTBX2/Y(+) (Atoh1-TBX2ME) mice. For generation of mTmG-positive controls Sox2CreERT2/+ males were mated with R26mTmG/mTmG females to obtain Sox2CreERT2/+;R26mTmG/+ mice, and Atoh1-CreERT2/+ males with R26mTmG/mTmG females to obtain Atoh1-CreERT2/+;R26mTmG/+ mice. Cre-negative littermates or Sox2CreERT2/+;R26mTmG/+ and Atoh1-CreERT2/+;R26mTmG/+ mice served as controls.<br><br>For embryonic staging, the detection day of a vaginal plug was defined as embryonic day (E) 0.5. To induce recombination, 4 mg of tamoxifen dissolved in corn oil were orally applied to timed pregnant or breast-feeding dams in a single pulse at E12.5, E15.5, E16.5 or P0-1. From E15.5 onwards, 2 mg of progesterone were additionally applied. Pregnant females and juvenile mice were euthanized by cervical dislocation, neonates were euthanized by decapitation. Embryos at E14.5, E16.5 and E18.5, neonates at P4 and juveniles at P17/21 were collected for analyses. Both sexes were interchangeably used with the exception of Sox2-TBX2ME mice, where we used male mice at embryonic stages and female mice at postnatal stages.
Wild animals	The study did not involve wild animals.
Field-collected samples	The study did not involve field-collected samples.
Ethics oversight	All animal work conducted for this study was performed in strict accordance to European and German legislation. The breeding and handling of mouse lines was performed at the central animal laboratory of the Hannover Medical School and approved by the Niedersächsisches Landesamt für Verbraucherschutz und Lebensmittelsicherheit (Permit Number: 33.12-42502-04-19/3081).

Note that full information on the approval of the study protocol must also be provided in the manuscript.
